# Poly-(ADP-ribose) serves as a scaffold for the methyltransferase METTL3/14 complex in the DNA damage response

**DOI:** 10.1093/nar/gkaf244

**Published:** 2025-04-12

**Authors:** Claudia Gonzalez-Leal, Jin Cai, Bram A F J de Groot, Andreas Wegerer, Julia Preisser, Martijn S Luijsterburg, Andreas G Ladurner

**Affiliations:** Department of Physiological Chemistry, Biomedical Center (BMC), Faculty of Medicine, LMU Munich, 82152 Planegg – Martinsried, Germany; International Max Planck Research School (IMPRS) for Molecules of Life, 82152 Planegg-Martinsried, Germany; Department of Physiological Chemistry, Biomedical Center (BMC), Faculty of Medicine, LMU Munich, 82152 Planegg – Martinsried, Germany; International Max Planck Research School (IMPRS) for Molecules of Life, 82152 Planegg-Martinsried, Germany; Department of Human Genetics, Leiden University Medical Center (LUMC), 2300 RC Leiden, The Netherlands; Department of Physiological Chemistry, Biomedical Center (BMC), Faculty of Medicine, LMU Munich, 82152 Planegg – Martinsried, Germany; Department of Physiological Chemistry, Biomedical Center (BMC), Faculty of Medicine, LMU Munich, 82152 Planegg – Martinsried, Germany; Department of Human Genetics, Leiden University Medical Center (LUMC), 2300 RC Leiden, The Netherlands; Department of Physiological Chemistry, Biomedical Center (BMC), Faculty of Medicine, LMU Munich, 82152 Planegg – Martinsried, Germany; International Max Planck Research School (IMPRS) for Molecules of Life, 82152 Planegg-Martinsried, Germany; Eisbach Bio GmbH, Am Klopferspitz 19, 82152 Planegg-Martinsried, Germany

## Abstract

PARP1, a crucial DNA break sensor, synthesizes poly-(ADP-ribose) (PAR), a nucleic acid that promotes the recruitment of DNA repair proteins. Emerging evidence highlights a role of RNA and RNA-binding proteins in DNA repair. Notably, the RNA–m^6^A methyltransferase complex METTL3/14 is implicated in repairing ultraviolet-induced DNA lesions. Here, we dissected the interplay between the two nucleic acids PAR and RNA and how METTL3/14 recruitment and m^6^A accumulation at laser-induced DNA lesions responds to PAR dynamics. *In vitro*, METTL3/14 recognized both PAR and RNA, yet PAR presence did not inhibit the methyltransferase complex’s catalytic activity. Acute knock-out of METTL3 rendered cells sensitive to transcription-blocking DNA damage and resulted in defects in transcription recovery and transcription-coupled DNA repair. Furthermore, combining METTL3 and PARP inhibitors led to an enhanced antiproliferative effect on cancer cells. Future therapeutic avenues may thus leverage the interplay between the nucleic acids PAR and RNA.

## Graphical Abstract

**Graphical Abstract:**
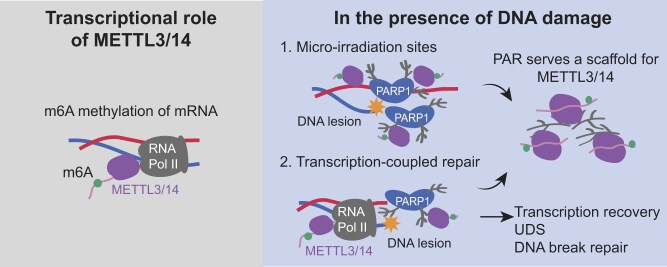

## Introduction

The DNA within our genome contains the essential instructions for the proper functioning of the cell, yet it constantly faces threats by ubiquitous endogenous and exogeneous sources of damage. To protect its integrity, cells have developed the DNA damage response (DDR), a network of pathways that repair compromised genomes. Central to this process is the poly-(ADP-ribose) polymerase 1 (PARP1), an early detector of DNA breaks, which upon binding to DNA ends catalyzes the formation of poly-(ADP-ribose) chains (PAR). The nucleic acid PAR acts in multiple ways: as a transient post-translational modification that modulates the activity of downstream targets, as a nucleic acid that is directly recognized through a variety of PAR-binding domains, as a polymeric anion that impacts chromatin structure or as a scaffold for DDR proteins [1]. Clinically, PARP inhibitors (PARPi) are used to treat certain cancers through synthetic lethality, for example, by exploiting genetic vulnerabilities present in BRCA1/2-deficient ovarian, breast, and prostate cancer [2].

The involvement of RNA in the DDR, including transcription-dependent and -independent roles [3] is increasingly recognized. Noncoding RNAs assist in the repair of double-strand DNA breaks, serving as template for homology-mediated repair, or aiding the compartmentalization of DNA lesions [4–6]. Additionally, RNA Polymerase II stalling upon collision with bulky DNA lesions acts as a surveillance mechanism, triggering the transcription-coupled nucleotide excision repair response [7, 8]. Furthermore, increased expression of DDR proteins after DNA damage supports a role for specific gene expression programs in the DNA damage stress response [9, 10].

Post-transcriptional modification of RNA provides an additional layer to modulate RNA metabolism. N^6^-methyladenosine (m^6^A), a prevalent RNA modification in eukaryotic cells, influences every stage of RNA metabolism (reviewed in [11]. The m^6^A deposition involves a holo-complex, with the heterodimer of the Methyltransferase-like-3 and -14 proteins (METTL3/14 complex) as methyltransferase subunit [12]. Structural and biochemical evidence indicate that METTL3 is the catalytic core that binds the cofactor SAM, while METTL14 serves as an RNA-binding scaffold and contributes to the catalytic center of the heterodimer [13–15]. M6A is recognized by YTH family proteins [16–18] and can be removed by the FTO and ALKBH5 demethylases [19, 20], making it a dynamic “epitranscriptomic” mark. Further, small molecule METTL3 inhibitors are also gaining increasing attention in cancer therapy [21] due to their potential to modulate cellular m^6^A functions.

The role of PARP1 extends to various aspects of RNA metabolism, from transcription initiation through chromatin remodeling [22, 23], interaction with transcription factors [24–27], regulation of RNA Polymerase II elongation [28, 29], and RNA processing [30–32]. Moreover, PARP1 can directly bind and modify RNA [33, 34], while DNA–RNA hybrids (R-loops) bind and activate PARP1 *in vitro* [35, 36]. PAR also can contribute to the formation of membrane-less sub-compartments enriched in RNA-binding proteins (RBPs) [37–39].

METTL3 mediates ultraviolet (UV) lesion repair [40] and R-loop resolution during DSB repair [41, 42], processes influenced by PAR dynamics, as evidenced by the impact of PARP inhibitors or the PARylation of intermediate factors. Moreover, *in vitro* evidence indicates that METTL3/14 mediates the methylation of damaged DNA [43, 44] and METTL3 was found to modify the long noncoding RNA NEAT1 in response to DNA damage, thereby affecting the function of the chromatin remodeler CHD4 [45]. This suggests a role for m^6^A in epigenetic regulation during the DDR. However, the nature of the interaction between the three nucleic acids PAR, RNA, and DNA, in the DDR and the role of the METTL3/14 complex require further analysis. Specifically, the mechanism underlying the METTL3/14 enrichment at sites of PAR activation during DNA damage is not fully understood.

In this paper, we investigated the timing and PAR dependence of m^6^A accumulation and METTL3/14 recruitment at UV lesions triggered by laser micro-irradiation. We assessed the binding of PAR and RNA to the METTL3/14 complex and found that while both nucleic acids are recognized by the complex, high concentrations of PAR displaced, but did not abolish RNA binding. Moreover, the presence of PAR did not significantly affect the catalytic activity of METTL3/14, suggesting that PAR serves as a scaffold for METTL3/14 recruitment and RNA modification. We further characterized the role of METTL3 in DNA repair and found that METTL3 depleted cells were sensitive to Illudin S. Using cells deficient in global-genome nucleotide excision repair (GG-NER), we observed reduced transcription recovery and unscheduled DNA synthesis (UDS) after UV irradiation. Furthermore, in the absence of METTL3, cells exhibited reduced efficiency repairing trabectedin-induced DNA lesions, observed as a reduction of γH2AX. Together, these data highlight a role of METTL3 in transcription-coupled NER (TC-NER). Our research also explored the potential interaction of METTL3 inhibitors with PARP inhibitors, highlighting how interfering with PAR dynamics may leverage the interplay between DNA repair and RNA modification.

## Materials and methods

### Cell culture

U2OS ΔMETTL3 and parental wild-type control cells were kindly provided by Yang Shi (Harvard Medical School) and described previously [40]. U2OS ΔPARP1 cells were previously characterized and described [46]. MOLM13 cells were obtained from DSMZ (ACC 554. DLD1 wild-type and DLD1 ΔBRCA2 cells were obtained from Horizon (HD PAR-008 and HD-105-007, respectively). MDA-MB-231 cells were obtained ATCC (HTB-26) and SUM149PT from BIOIVT. U2OS and MDA-MB-231 cells were maintained in Dulbecco’s modified Eagle’s medium (DMEM) (1 g/l glucose; Sigma), supplemented with 10% fetal bovine serum (FBS) (Gibco) and 1% penicillin/streptomycin. MOLM13 and DLD1 cells were maintained in RPMI-1640 media (Gibco) supplemented as described for DMEM media, and SUM149PT cells were maintained in Ham’s F12 supplemented media. All cells were cultured at 37°C in a humidified incubator with 5% CO_2_ and were routinely tested for mycoplasma by polymerase chain reaction (PCR)-based testing.

### Plasmids and DNA transfection

FLAG-METTL3, FLAG-METTL14 and FLAG-WTAP were purchased from Addgene (#53739, #53740, and #53741, respectively). FTO was amplified from U2OS wild-type complementary DNA. These proteins were subcloned into EGFP-C1 or pmCherry-C1 vectors (Clontech) using conventional restriction enzyme cloning. GFP-PARP1 WT [45], mCherry-PARP1 WT [47] and pmCherry-PARP1 E998K [48] was previously described. The mCherry-LacR-macroH2A.1.1 used for co-localization assays was described [49].

mCherry-LacR-METTL14 was subcloned from FLAG-METTL14 using following primers:

fw 5-GAGCTCAAGCTTCGAATTCTGATAGCCGCTTGCAGGAG-3

rv 5-CGCGGTACCGTCGACTGCTTATCGAGGTGGAAAGC-3

LacR-METTL14 was subcloned from mCherry-LacR-METTL14 using following primers:

fw 5-GCTACCGGTCGCCACCATGGTGAAACCAGTAACG-3

rv 5-CGCGGTACCGTCGACTGCTTATCGAGGTGGAAAGC-3

METTL3 and METTL14 truncation mutants were generated using the following primers:

*METTL3 N-construct (2–219)*

fw 5-GGACGAGCTGTACAAGTCCGGATCGGACACGTGGAGCTC-3

rv 5-CGAAGCTTGAGCTCGAGATCTGAGTTATGAGGCAGCATGTTTCC-3

*METTL3 Mid-construct (198–352)*

fw 5-GGACGAGCTGTACAAGTCCGGACTGAACTCTTCAGCATCGGA-3

rv 5-TCGAAGCTTGAGCTCGAGATCTGAGTTACTCCTGGCTTGGCG-3

*METTL3 C-construct (351–580)*

fw 5-CGAGCTGTACAAGTCCGGACAGGAGCTTGCTCTTACACAG-3

rv 5-CGAAGCTTGAGCTCGAGATCTGAGTTATAAATTCTTAGGTTTAGAGATG-3

*METTL14 ΔRGG (2–402)*

fw 5-ATATATCTCGAGCGGATAGCCGCTTGCAGG-3

rv 5-ATATATAGGATCCTTAGGGAGGAGGCG-3

All plasmids were transfected into U2OS cells with XtremeGene HP DNA transfection reagent (Sigma) following manufacturer’s instructions.

### Generation of cells expressing wild-type GFP-METTL3

Cells stably expressing GFP-METTL3 WT were generated by transfecting U2OS ΔMETTL3 cells with the desired vector. Cells were then selected with 500 μg/ml G418 for 2 weeks. Cells were screened for the expression of the construct using western blot. To sustain expression, cells were maintained with 200 μg/ml G418 while in culture.

### Generation of XPC knockout cells

Parental RPE1-hTERT cells stably expressing inducible Cas9 (iCas9) that are also knockout for *TP53* and the puromycin-N-acetyltransferase *PAC1* gene were described previously (referred to as RPE1-iCas9) [50]. RPE1-iCas9 cells were co-transfected with Cas9-2A-EGFP (pX458; Addgene #48138) together with two pU6-sgXPC-PGK-puro-2A-tagBFP plasmids each encoding different XPC guide RNAs from the LUMC/Sigma–Aldrich single guide RNA (sgRNA) library using lipofectamine 2000 (Invitrogen). The used sgRNAs are 5-CCGAAGATATGTCTCAAACTCCA-3 (exon 5) and 5-TGGGGGTTTCTCATCTTCAAAGG-3 (exon 2). Cells were FACS sorted on EGFP/tagBFP and plated at low density, after which individual clones were isolated, expanded, and verified by western blot analysis and/or Sanger sequencing. RPE1-iCas9 XPC-KO clone 5 was used for further analysis.

### Lentiviral transduction

The pLenti-guide-mCherry plasmid was a gift from Sylvie Noordermeer (LUMC) [51]. Overlap PCR was used to introduce an optimized sgRNA scaffold (OSS) sequence based on [52] into pLenti-guide-mCherry using the following primers:

**Table:** 

fw-Lenti-OSS-0	5-CAATCAATTAAAATTGGTCCTGTTTGATTTCCATC-3
rv-Lenti-OSS-0	5-CAAAGATTCCTCTCTGTTTAAAACTTTATCCATCTTTG-3
fw-Lenti-OSS-1	5-CTCTGTTTAAGAGCTATGCTGGAAATAGCAAG-3
rv-Lenti-OSS-1	5-CTTGCTATTTCCAGCATAGCTCTTAAACAGAG-3
fw-Lenti-OSS-2	5-CTATGCTGGAAACAGCATAGCAAGTTTAAATAAGG-3
rv-Lenti-OSS-2	5-CCTTATTTAAACTTGCTATGCTGTTTCCAGCATAG-3

The overlap PCR product was inserted into pLenti-guide-mCherry as an NsiI/XmaI fragment to generate pLenti-guide-OSS-mCherry, which was confirmed by sequencing. The sgRNAs targeting METTL3 (5-TCTGAACCAACAGTCCACTA-3), CSB (5-AGACAGAATGATCCGATGAG-3), or CSA (5- GATGTTGAAAGAATCCACGG-3) were inserted into pLenti-guide-OSS-mCherry by digestion the vector with BsmBI and inserting annealed oligonucleotides:

**Table:** 

fw-sgMETTL3	5-CACCGTCTGAACCAACAGTCCACTA-3
rv-sgMETTL3	5-AAACTAGTGGACTGTTGGTTCAGAC-3
fw-sgCSB	5-CACCGAGACAGAATGATCCGATGAG-3
rv-sgCSB	5-AAACCTCATCGGATCATTCTGTCTC-3
fw-CSA	5-CACCGGATGTTGAAAGAATCCACGG-3
rv-CSA	5-AAACCCGTGGATTCTTTCAACATCC-3
fw-LUC	5-CACCGGGCGCGGTCGGTAAAGTTGT-3
rv-LUC	5-AAACACAACTTTACCGACCGCGCCC-3

For lentiviral particle production, HEK293T cells (ATCC CRL-3216) were transfected with vectors pLenti-sgCSB-OSS-mCherry or pLenti-sgMETTL3-OSS-mCherry, VSV-G, RRE, and REV using PEI (Sigma–Aldrich) in OPTI-MEM (Gibco) containing 10% fetal calf serum (FCS). The medium (DMEM (Gibco), 10% FCS, 1% Penicillin-Streptomycin (P/S) and 10 mM HEPES, pH 7.6 (Gibco)] was refreshed after 24 h. The virus-containing supernatant was then collected 24 h after medium change and filtered with a 0.44-μm filter. RPE1-iCas9 XPC-KO clone 5 cells were transduced with 500 μl lentiviral particles in the presence of 4 μg/ml polybrene (Sigma–Aldrich) and 10 mM Hepes pH 7.6. After 24 h, the medium was changed [DMEM, 10% FCS, 1% P/S, and 2 μg/ml doxycycline (Sigma–Aldrich)] to prevent re-transduction and activate Cas9 expression. Cells were subsequently left for 5 days to ensure efficient depletion at the protein level. At least 90% of the cells were transduced based on flow cytometric analysis of mCherry expression.

### Fluorescent imaging of U2OS cells

For live-cell imaging, cells were seeded in eight-well Nunc Lab-Tek chambers (Thermo Fischer Scientific) and recorded while in Leibovitz’s L-15 media supplemented with 10% FBS at 37°C and no CO_2_. All imaging experiments were performed on a Zeiss AxioObserver Z1 confocal spinning-disk microscope with a sCMOS ORCA Flash 4.0 camera (Hamamatsu). A C-Apo 63× water immersion objective lens was used for capturing live-cell imaging experiments, while a 40× C-Apochromat/1.2 Korr water objective was used for the analysis of fixed cells.

### Live-cell imaging and quantification of laser micro-irradiation

Cells were seeded, transiently transfected 48 h before imaging and sensitized by incubating with 1 μM BrdU for 16 h. Cells with low-comparable transgene expression levels were selected for analysis. DNA damage was induced through a line of 88 pixels using 10% power of a 355 nm laser operated through a single-point scanning head (UGA-42 firefly, Rapp OptoElectronics) with 20–22 runs per object. Images were recorded using for 5 min following irradiation recorded at intervals of 5 s. For treatments, cells were incubated as follows: 1:500 dimethyl sulfoxide (DMSO) for 1 h, 1 μM olaparib (PARPi; SelleckChem) for 1 h, 1 μM PDD00017273 (PARGi; Sigma) for 1 h, 2 μg/ml α-Amanitin (Sigma) for 4 h, and 0.5 μg/ml Actinomycin D (Applichem) for 2 h. A custom macro in Fiji/ImageJ was used to measure the accumulation of fluorescently tagged proteins at micro-irradiation sites. The images are registered using the StackReg plugin [53], a background control outside of the cell was selected with a 30 × 30 pixels selection, and the region-of-interest (ROI) with a 25 × 100 pixels selection. Nuclear signal was used as nuclear control, which was automatically selected using the Li auto-threshold algorithm [54]. The recruitment was then calculated as the ratio of the mean fluorescent intensities of the ROI over the nuclear signal, corrected for background signal and relative to the mean fluorescent intensity before micro-irradiation.

### m^6^A accumulation after micro-irradiation

Cells were prepared and micro-irradiated as for live-cell imaging. Following irradiation, the cells were incubated for the indicated times, fixed and stained as described [55]. In brief, samples were washed with ice-cold phosphate-buffered saline (PBS), fixed with 2% paraformaldehyde in PBS + 0.1% Triton X-100 for 15 min at room temperature and permeabilized with PBS + 0.1% Triton X-100 for 2 × 10 min at room temperature. To denature the DNA, samples were washed with PBS + [PBS + 0.5% bovine serum albumin (BSA) + 0.15% glycine] and incubated for 5 min with 0.07 M NaOH. After washing with PBS +, the cells were incubated with diluted primary antibody overnight at 4°C. Unbound antibody was removed by washing 5× with PBS + 0.1% Triton and 2× with PBS +. Samples were incubated for 1 h at room temperature in the dark with Alexa Fluor 488/568-conjugated fluorescent antibody (Thermo Fisher Scientific) and Hoechst 33 342 (Thermo Fischer Scientific) diluted 1:2000 and 1:1000 in PBS + respectively. Unbound antibody was removed by washing 5× with PBS + 0.1% Triton and 2× with PBS +.

Antibodies: mouse-*anti*-CPD (1:1000, Cosmo Bio, CAC-NM-DND-001), rabbit-anti-m^6^A (1:1000, Synaptic Systems, 202003), goat-anti-Mouse IgG-Alexa Fluor™ 488 (1:2000, Thermo Fisher Scientific, A-11001), donkey-anti-Rabbit IgG-Alexa Fluor™ 568 (1:2000, Thermo Fisher Scientific, A-10042).

### LacO-LacR system for detecting PARylated and PAR-interacting proteins

LacO-LacR experiments were performed as described in [49]. U2OS 2–6-3 cells with repetitive copies of the LacO-containing cassette were plated into eight-well Labteck. Cells were co-transfected the next day either with mCherry-LacR-macroH2A.1.1 and the GFP-fused protein of interest, or with LacR-METTL14, mCherry-METTL3, and GFP-PARP1, and sensitized by incubating with 1 μM BrdU for 16 h. Prior to irradiation, the cells were treated for 1 hr with 1 μM PARGi, followed by 1 h with 1 μM olaparib when indicated. Micro-irradiation was combined with live-cell imaging as described and accumulation at the LacO site was quantified in ImageJ, correcting for nuclear signal outside the foci.

### Immunofluorescence staining

For all stainings after Ultraviolet-C (UVC) irradiation, cells were grown in coverslips and irradiated with 10 J/m2. For 6–4 PPs staining, cells were fixed with 4% paraformaldehyde in PBS, washed 3× with PBS, and permeabilized for 5 min with PBS + 0.5% Triton X-100 on ice. After washing 3× with PBS, samples were denatured with 2 M HCl for 30 min at room temperature. Samples were washed 5× with PBS and blocked with PBS + 20% FBS for 1 h at room temperature while gentle tilting. Next, samples were washed with 5× with PBS and incubated with primary antibody diluted 1:300 in PBS + 5% FBS for 1 h. After washing 5× with PBS, samples were incubated with secondary antibodies as described before. Samples were washed 5× with PBS and mounted as described. For cyclobutene pyrimidine dimer (CPD) staining samples were processed as described for m^6^A staining. For ATF3 staining, samples were fixed for 15 min with 2% paraformaldehyde (PFA) in PBS + 0.2% Triton X-100 and permeabilized for 3× short washes and 2 × 10 min washes with 0.1% Triton X-100 in PBS. Samples were blocked with 5% BSA in PBS with 0.1% Tween for 30 min at room temperature, and primary was diluted 1:500 in 1% BSA in PBS + 0.1% Tween and incubated overnight at 4°C. Next, samples were washed for 10 min 2× with 0.1% Triton X-100 in PBS, and 1× with 1% BSA in PBS with 0.1% Tween. Secondary antibody was diluted in 1% BSA in PBS with 0.1% Tween and incubated for 1 h at room temperature (RT) in the dark. Samples were washed 2 × 10 min in 0.1% Triton X-100 in PBS, and 1× in 1% BSA in PBS with 0.1% Tween before mounting using Aqua-poly mount (Polysciences Inc).

Antibodies: mouse-*anti*-6–4PP (1:300, Cosmo Bio, NM-DND-002), rabbit-*anti*-ATF3 (1:1000, Abcam, ab207434), mouse-*anti*-CPD (1:1000, Cosmo Bio, CAC-NM-DND-001), rabbit-anti-m^6^A (1:1000, Synaptic Systems, 202 003), mouse-anti-PAR (1:1000, RCB, RCB1142), goat-anti-Mouse IgG-Alexa Fluor™ 488 (1:2000, Thermo Fisher Scientific, A-11001), donkey-anti-Rabbit IgG-Alexa Fluor™ 568 (1:2000, Thermo Fisher Scientific, A-10042).

### Nascent RNA imaging

Cells were seeded in coverslips in DMEM supplemented with 1% FBS, exposed to UVC irradiation (10 J/m2), and allowed to recover in DMEM supplemented with 10% FBS for the desired times. After incubation, cells were incubated in DMEM supplemented with 0.5 mM ethynyl-uridine (EU) for 2 h at 37°C, washed with PBS and fixed for 15 min with 4% paraformaldehyde in PBS. Samples were rinsed 2× with 3% BSA in PBS and permeabilized for 20 min with PBS + 0.5% Triton X-100. Click-iT reaction to couple secondary antibody was performed using the Click-iT^®^ RNA Alexa Fluor^®^ 594 Imaging Kit (Thermo Fischer Scientific). Samples were rinsed 2× with 3% BSA in PBS, followed by 2× PBS and mounted as described before.

### Incision assay (γH2AX after trabectedin)

Cells were either treated with 10 μM PARPi (olaparib; KU-0059436, Bio-Connect), 10 μM NEDD8i (MLN4924, Sigma–Aldrich), or nontreated 1 h before treatment. Cells were then treated with 10 nM trabectedin (MedChemExpress) for 4 h. During the last 15 min, 20 μM 5-ethynyl-2′-deoxyuridine (5-EdU; Jena Bioscience) was added. Cells were then washed with 300 mM sucrose (Merck) in PBS on ice, pre-extracted with 300 mM sucrose and 0.25% Triton X-100 in PBS for 2 min on ice and fixed with 3.7% formaldehyde in PBS for 15 min at RT. Cells were then permeabilized with 0.5% Triton X-100 in PBS for 10 min at RT, and blocked in 3% BSA (Thermo Fisher) in PBS. Dividing cells were visualized by click-it chemistry, labeling the cells for 30 min with a mix of 60 μM Atto azide-Alexa647 (Atto Tec), 4 mM copper sulfate (Sigma), and 10 mM ascorbic acid (Sigma) in a 50 mM Tris-buffer. After washing with PBS, cells were blocked with 100 mM glycine (Sigma) in PBS for 10 min at RT and subsequently with 0.5% BSA and 0, 05% Tween-20 in PBS for 10 min at RT. To visualize γH2AX, cells were incubated with a primary antibody for phospho-Histone H2A.X Ser139 (JBW301, Merck) for 2 h at RT and then with a secondary antibody Anti-Mouse Alexa 488 (A-11029, Thermo Fisher) and 4'-6'-diamidino-2-phenylindole (DAPI) for 1 h at RT and mounted in Polymount (Brunschwig).

### RNA recovery of RNA synthesis

Transduced RPE1 cells deficient in global genome nucleotide excision repair (GGR) (XPC-KO cells) cells were starved for 24 h (DMEM, 1% FCS) and successively irradiated with UVC light (12 J/m^2^), allowed to recover for the indicated periods, and pulse-labeled with 400 μM EU (Jena Bioscience) for 1 h followed by a 15 min medium-chase with DMEM without supplements. Cells were either treated with 10 μM PARPi (olaparib; KU-0059436, Bio-Connect), 10 μM NEDD8i (MLN4924, Sigma–Aldrich), or nontreated 1 h before irradiation with UVC. Cells were fixed with 3.7% formaldehyde in PBS for 15 min, permeabilized with 0.5% Triton X-100 in PBS for 10 min at room temperature, and blocked in 1.5% BSA (Thermo Fisher) in PBS. Nascent RNA was visualized by click-it chemistry, labeling the cells for 1 h with a mix of 60 μM Atto azide-Alexa647 (Atto Tec), 4 mM copper sulfate (Sigma), 10 mM ascorbic acid (Sigma), and 0.1 μg/ml DAPI in a 50 mM Tris-buffer (pH 8). Cells were washed extensively with PBS and mounted.

### TCR-specific unscheduled DNA synthesis (TCR-UDS)

Transduced RPE1 cells deficient in GGR (XPC-KO cells) were used to specifically measure UDS by TC (TCR-UDS) [56]. Cells were plated in DMEM supplemented with 10% FCS. Subsequently, cells were placed in DMEM without FCS for 24 h prior to UV irradiation to reduce the excess of available deoxy-thymidine in the culture medium. Cells were either treated with 10 μM PARPi (olaparib; KU-0059436, Bio-Connect), 10 μM NEDD8i (MLN4924, Sigma–Aldrich), or nontreated 1 h before irradiation with UVC. Cells were locally UV irradiated through 5 μm pore filters (Millipore) with 100 J/m^2^ and immediately pulse-labeled with 20 μM 5-ethynyl-deoxy-uridine (EdU; VWR) and 1 μM FuDR (Sigma Aldrich) for 4 h. After medium-chase with DMEM containing 10 μM thymidine for 15 min, cells were fixed with 3.7% formaldehyde in PBS for 15 min at room temperature and stored in PBS. Next, cells were permeabilized for 20 min in PBS with 0.5% Triton X-100, washed two times with PBS + 3% BSA (Thermo Fisher) and washed one time in 1× PBS. The incorporated EdU was visualized by click-it chemistry, labeling the cells for 30 min with a mix of 60 μM atto azide-Alexa647 (Atto Tec), 4 mM copper sulphate (Sigma) and 10 mM ascorbic acid (Sigma) in a 50 mM Tris-buffer (pH 8). After this, the cells were post-fixed with 2% PFA for 10 min and blocked with 100 mM glycine for 10 min at room temperature. Cells were washed extensively with PBS, DNA was denatured with 0.5 M NaOH for 5 min, blocked with 10% BSA (Thermo Fisher) in PBS for 15 min, and equilibrated in 0.5% BSA and 0.05% Tween-20 in PBS (Wash buffer: WB). Sites of local UV damage were visualized by labeling the cells for 2 h at room temperature, with mouse anti-CPD (Cosmo Bio; 1:1000 in WB). After primary antibody incubation, cells were washed extensively with WB, stained with goat anti-mouse IgG-Alexa488 (1:1000 in WB) for 1 h, again washed extensively with WB, stained with 0.1 μg/ml DAPI, washed extensively with PBS, and mounted.

### Microscopic analysis of fixed RPE1 cells

Images of fixed samples were acquired on a Zeiss AxioImager M2 widefield fluorescence microscope equipped with 63× PLAN APO (1.4 NA) oil-immersion objectives (Zeiss) and an HXP 120 metal-halide lamp used for excitation. Fluorescent probes were detected using the following filters for DAPI (excitation filter: 350/50 nm, dichroic mirror: 400 nm, emission filter: 460/50 nm), Alexa 488 (excitation filter: 470/40 nm, dichroic mirror: 495 nm, emission filter: 525/50 nm), or Alexa 647 (excitation filter: 640/30 nm, dichroic mirror: 660 nm, emission filter: 690/50 nm). Images were recorded using ZEN 2012 (blue edition, version 1.1.0.0) and analyzed in ImageJ (1.47v-1.48v). EdU and CPD intensities for the TCR-UDS were quantified by a previously published protocol and macro [57]. EU, EdU, and γH2AX (background) intensities for the Incision assay and RNA recovery synthesis (RRS) were quantified using a custom designed ImageJ macro. Nuclear intensities of EU, EdU, or γH2AX were quantified by subtracting the average (median RRS) intensities outside the nucleus from the average intensity inside the nucleus. S-phase positive cells for the incision assay were selected based on their EdU intensity. Graphs were plotted and analyzed using Graphpad Prism 8 (v8.4.2), Microsoft Excel 365, and Adobe Illustrator 2022.

### CellTiter-Glo^®^ 2.0-based cell viability assay

Transduced RPE1 cells were seeded (in duplicate) in a 96-well plate containing different numbers of cells based on their expected sensitivity to Illudin S. Cells were plated in DMEM supplemented with 10% FCS and an increasing dose of Illudin S (Santa Cruz; [0, 100, 200, and 400 pg/ml] to determine the cell viability in various knock-out backgrounds. Cells were allowed to grow for 7 days after which their sensitivity to Illudin S was determined, based on the cell viability, via a CellTiter-Glo^®^ 2.0 (Promega) assay according to the manufacturers protocol. Signal intensity was corrected for the DMEM medium background luminescence levels by measuring empty wells supplemented with DMEM with 10% FCS. The corrected Luminance signal was calculated by subtracting the average background from the luminescence signal of each sample. To calculate the relative viability, the average luminescence signal (per duplicate) was divided by the signal of the nontreated control of the individual condition and multiplied by the fraction of cells. Graphs were plotted and analyzed using Graphpad Prism 8 (v8.4.2), Microsoft Excel 365, and Adobe Illustrator 2022.

### Western blot analysis of acute KO pools in RPE1 cells

Transduced RPE1 cells extracts were obtained by cell lysis and boiled for 15 min at 96°C in Laemmli buffer. Afterwards, proteins were separated by sodium dodecyl sulfate–polyacrylamide gel electrophoresis on 4%–12% Criteron™ XT Bis-Tris pre-cast gels (Biorad) in NuPAGE™ 3-(*N*-morpholino)propanesulfonic acid (MOPS) sodium dodecyl sulfate running buffer (Thermo Fisher) and transferred to 0.45 μm PVDF membranes (Immobilon). Polyvinylidene fluoride (PVDF) membranes were blocked for 1 h using a PBST [1× PBS, 0.1% Tween-20 (Sigma)] 5% milk (Campina, milk powder) solution. Protein abundance was determined by immunoblotting with a primary antibody [α-CSB; 10R-1587 – Fitzgerald (1/1000), α-CSA; ab137033 – Abcam (1/500), α-METTL3; #A8370 – Abclonal (1/1000)] for 2 h followed by a secondary antibody (CF770 Goat Anti-Mouse IgG; #20077 – VWR, CF680 Goat Anti-Rabbit IgG; #20067 – VWR) for 1 h both at room temperature in PBST 5% milk solution. Protein presence was detected using the Odyssey infrared imaging scanning system (LI-COR biosciences, Lincoln, Nebraska USA).

### CAS9-mediated editing efficiency

Genomic DNA (gDNA) from transduced RPE1 cell extracts was obtained by cell lysis in 1× WCE buffer (50 mM KCl, 10 mM Tris, pH 8.0, 25 mM MgCl_2_, 0.1 mg/ml gelatin, 0.45% Tween-20, 0.45% NP-40) containing 0.1 mg/ml Proteinase K (EO0491; Thermo Fisher Scientific) at 56°C for 2 h followed by buffer inactivation at 96°C for 10 min. After crude gDNA extraction 1 μl of the lysate was used to amplify the gDNA approximately 1 kb flanking the guide RNA target site of METTL3 (FW 5-tgttcaaactcacgcactag-3, RV 5-tgagttagagaagaagttgc-3). The flanking regions were amplified using a Taq Polymerase Mastermix (Qiagen) with a regular PCR. Afterwards, 1 μl of the first amplicon was used as template for a nested-PCR to gain higher specificity (FW 5-cattcatctccctatatttc-3, RV 5-cttattttcagttcacggtc-3). The final PCR product was sent for sanger-sequencing (Macrogen Europe). Editing efficiency was determined and calculated using the ICE CRISPR analysis tool from Synthego (https://ice.synthego.com/) by suppling a wild-type and iCAS9 mutated amplicon together with the gene targeting guide (METTL3: 5-TCTGAACCAACAGTCCACTA-3).

### siRNA-mediated gene knockdowns

Cells were transfected twice with 10 nM of siRNA using Lipofectamine RNAiMAX (Invitrogen) following manufacturer’s protocol, with the second transfection taking place 6 h after the first one. Experiments were performed 48 h after treatment.

### Cell viability assay and drug interaction

Effects of olaparib and STM2457 was assessed as following: 1000 MOLM-13 or 5000 MDA-MB-231, SUM149PT, or DLD1 cells were seeded per well one day before treatment in SCREENSTAR 96 well plates (Greiner Bio-One) using phenol-red free media. Next day, cells were treated following a 2D titration scheme with increasing concentrations of each individual drug as indicated. Cell viability was measured 72 h after treatment using the CellTiter-Glo^®^ Luminescent Cell Viability Assay (Promega) according to manufacturer’s instructions. Luminescence was measured using a Tecan Infinite M1000 PRO The percentage of inhibition was calculated as:


\begin{eqnarray*}
&& \% \ {\rm inhibition} =\nonumber\\ && \left( {1 - \frac{{{\rm Luminescence\ of\ sample} - {\rm All\ dead\ sample}}}{{{\rm All\ alive\ sample} - {\rm All\ dead\ sample}}}} \right)*100
\end{eqnarray*}

All dead and all alive samples correspond to DMSO conditions in which all cells were dead or viable correspondingly. Drug interaction was assessed using the SynergyFinder 2.0 online tool [58] using the highest single agent (HSA) model. For each cell line, data represents at least three biological replicates with two technical replicates. Values shown as mean ± standard error of the mean (SEM).

### Purification of PAR

PAR chains were purified following a published protocol [59]. Briefly, a PARylation reaction of purified PARP1 lacking the HD domain was carried out in 50 mM Tris–HCl, pH 8.0, 4 mM MgCl_2_, 20 mM NaCl, 5 mM NAD^+^, 250 μM dithiothreitol (DTT), salmon sperm DNA, and 150 nM human PARP1 at 37°C for 60 min. To stop the reaction, cold TCA (20% w/v) was added and incubated for 15 min. The reaction mix was centrifuged for 10 min at 9000 × *g*, 4°C and rinsed, with ice-cold 99.8% ethanol and incubated for 10 min on ice. The reaction mix was centrifuged, washed with ice-cold ethanol again before centrifuging at 9000 × *g* for 20 min. Ethanol was removed and the pellet was air-dried. The cleave the polymer, samples were treated with 0.5 M KOH/50 mM ethylenediaminetetraacetic acid (EDTA) for 10 min at 37°C in a shaking water bath. Tris–HCl was adjusted to 100 mM and pH was adjusted to 8.0. Fifty millimolar MgCl_2_ and 110 μg/ml of DNAse I were added and incubated for 2 h at 37°C while shaking. Then, 220 μg/ml of Proteinase K and 1 mM CaCl_2_ were added and digestion was carried out overnight. PAR polymer was purified using phenol-chloroform extraction. PAR was resuspended in water and concentration was estimated based on the absorption coefficient of mono-(ADP-ribose) monomers measured at 258 nm and using the Lambert–Beer equation: [PAR] = A258 nm cm^−1^/13 500 cm^−1^M^−1^.

### Thermal shift assay

Prometheus NT.48 (NanoTemper GmbH, Munich, Germany) was used to perform dye-free thermal shift assays (TSAs). Capillaries were filled with 10 μl of binding mix containing 1 μM of recombinant protein and increasing concentrations of ligand in 20 mM Tris pH 7.5 buffer supplemented with 50 μM ZnCl_2_, 1% glycerol, and 1 mM DTT. The excitation light was adjusted to get readings above 2000 arbitrary units and samples were measured in a temperature range of 20–80°C with a temperature slope of 1°C/min. Three technical replicates were analyzed per run, scattering information was used to exclude aggregate formation. For visualization, data was normalized to min and max values of the first derivative of the 350/330 nm ratio. T_m_ values were obtained from the NT.48 Software.

### Electrophoretic mobility shift assay

Purified METTL3 and 20 ng of Cy3-labeled RNA were incubated in 25 μl binding reaction buffer (40 mM KCl, 20 mM Tris pH 7.6, 1.5 mM MgCl_2_, 0.5 mM ethylene glycol-bis(ß-aminoethyl ether)-N,N,N',N'-tetraacetic acid (EGTA), 10% glycerol, 200 ng/μl BSA, 1 mM DTT supplemented with 1 U/μl of RiboLock) for 15 min at 26°C. For competition assay, different amount of PAR was further incubated with this reaction suspension for 75 min at 26°C. Samples were separated on 6% native polyacrylamide gel (pre-run at constant current of 80 V for 45 min) in 0.4× TBE buffer (TBE buffer: 90 mM Tris, 90 mM boric acid and 2 mM EDTA) at a constant current of 80 V for 70 min. Gels were imaged using a ChemiDoc MP (Bio-Rad).

### Microscale thermophoresis

We used Monolith NT.115 (NanoTemper GmbH, Munich, Germany) to assess the binding of recombinant METTL3/14 to fluorescently tagged RNA and PAR. Capillaries were filled with 10 μl of binding mix containing 100 nM of RNA or PAR probe and increasing concentrations of recombinant protein in 20 mM Tris pH 7.5 buffer supplemented with 50 μM ZnCl_2_, 1% glycerol, and 1 mM DTT. The excitation light was adjusted to get readings above 2000 arbitrary units. Two technical replicates were analyzed per run and quality of the traces was used to exclude anomalies such as aggregates or air bubbles. Data was analyzed using the NT Analysis Software and *K*_D_ values were calculated by fitting a Hill slope model in GraphPad Prism8. Accuracy of the model is reported as R^2^ value for each condition.

### Western blotting

Samples were resuspended in Laemmli buffer, run on 4%-20% gradient gels (Bio-Rad) and blotted for 25 min at 15 V, 1.5 A using the Trans-Blot Turbo Transfer System (Bio-Rad). Blots were blocked for 30 min in TBS-Tween (0.05%) + 5% skim milk. Blots were washed 3 × 5 min with TBS-Tween and stained with primary antibodies overnight at 4°C. Blots were washed 3 × 5 min with TBS-Tween and incubated for 1 h with secondary antibodies (CF 680 or 770; Sigma, or IgG-rabbit or mouse HRP; Bio-Rad) diluted in TBS-Tween. After washing 3× with TBS-Tween, and 1× with TBS, blots were imaged using an Odyssey CLx (Li-Cor) or a ChemiDoc MP Imaging System (Bio-Rad).

Antibodies: rabbit-*anti*-ATF3 (1:1000, Abcam, ab207434), mouse-anti-Tubulin (1:10 000, Sigma, T9026), rabbit-*anti*-METTL3 (1:1000, ABclonal, A8370), mouse-*anti*-METTL14 (1:1000, Abcam, ab220030), *anti*-PARP1 (1:10 000, homemade, N/A), mouse-*anti*-RPB1 (1:1000, Active Motif, 39 097), rabbit-*anti*-RPB1 pS2 (1:1000, Abcam, ab5095), mouse-*anti*-XPA (1:1000, Thermo Fischer Scientific, MA5-13835), mouse-*anti*-XPC (1:1000, Santa Cruz Biotechnology, sc-74410), rabbit-*anti*-CSB (1:1000, Thermo Fischer Scientific, PA579218), goat-*anti*-GFP (1:1000, homemade, N/A), mouse-CF 680 (1:10 000, Sigma, SAB4600205), rabbit-CF 680 (1:10 000, Sigma, SAB4600200), mouse-CF 770 (1:10 000, Sigma, SAB4600214), rabbit-CF 750 (1:10 000, Sigma, SAB4600375), goat-*anti*-GFP (1:1000, homemade, N/A).

### Quantification and statistical analysis

Data processing was performed using Microsoft Excel, while Prism GraphPad was used for data visualization and statistical analysis. All experiments were performed in two to four independent replicates with at least two technical replicates. To compare multiple conditions, we used one-way Analysis of Variance (ANOVA), with Turkey’s or Dunnett’s *post-hoc* test to compare all means against each other with the former, or all means against control with the later. For cell cycle analysis, a two-way ANOVA with Turkey’s *post-hoc* test was used to compare differences between genomic background, treatment, and cell cycle phase. Significance levels were defined as follow: ns *P* > .033, * *P* < .033, **P < .002, *** *P* < .001, **** *P*< .0001. Each figure legend details the number of assayed cells and replicates analyzed, how the data are represented [mean ± SEM or standard deviation (SD)], and the statistical test used. The significance between conditions is displayed in the figures as lines connecting the compared pair.

## Results

### PAR drives the accumulation of m^6^A at DNA damage sites

**Figure 1.**
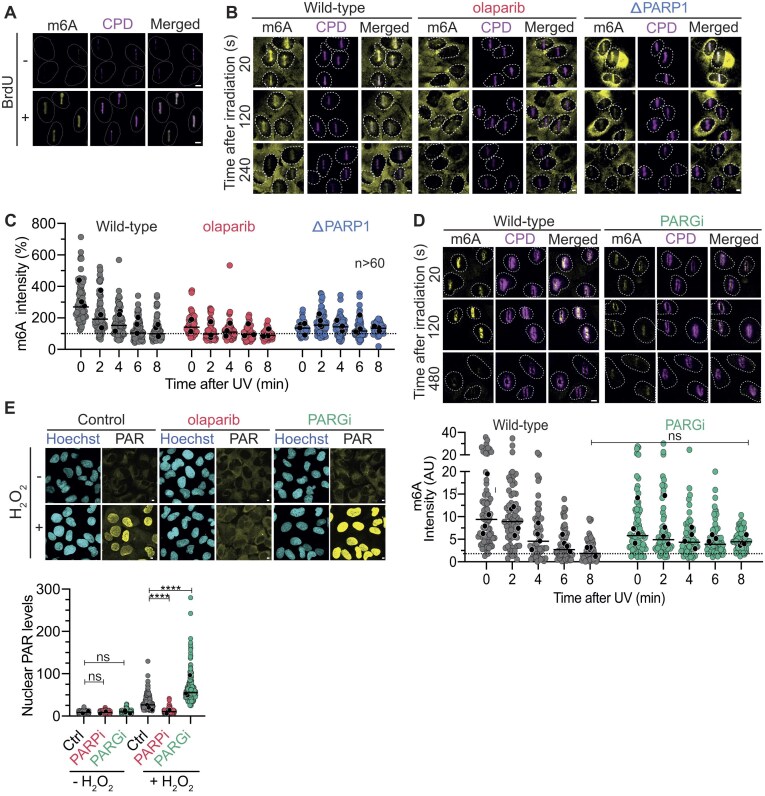
PAR drives the accumulation of m^6^A at micro-irradiation sites. (**A**) Representative images of m^6^A immunofluorescence signal after micro-irradiation in U2OS cells with or without BrdU. (**B**) Representative images of m^6^A signal after micro-irradiation in BrdU pre-sensitized cells, with or without the PARP inhibitor olaparib and in PARP1 deficient (ΔPARP1) cells. (**C**) Quantification of m6A immunofluorescence signal after micro-irradiation; 125–143 cells were analyzed per condition. (**D**) (*Top*) Representative images and (bottom) quantification of m^6^A signal after micro-irradiation in BrdU pre-sensitized cells, with or without PARG inhibitor. (**E**) (*Top*) Representative images and (*bottom*) quantification of PAR immunofluorescence signal after H_2_O_2_ with or without olaparib or PARGi treatment. More than 300 cells were analyzed per condition. For panels (C), (D), and (E), all nuclei from three independent experiments are depicted as individual points. The means of three biological replicates are depicted as black points, while the bar represents the median of all points. Conditions were compared using one-way ANOVA using Tukey’s test to compare each treatment against its control, **** *P*< .0001. The scale bar is 5 μm for all images.

To characterize the molecular mechanism that leads to the accumulation of m^6^A at UV-induced DNA damage sites, we used UV micro-irradiation experiments followed by immunofluorescence staining. This validated that m^6^A rapidly and transiently accumulates at DNA lesions in BrdU pre-sensitized cells (Fig. 1A). In our experimental setup, m^6^A was detectable at DNA lesions within seconds following laser irradiation and diminished within the first 6 min (Fig. 1B and C). This rapid accumulation of m^6^A mirrored the kinetics of PARylation [48], a critical early DDR modification. Consistent with prior studies [40], we found that treatment of cells with the PARPi olaparib abolished the accumulation of m^6^A at UV-induced DNA lesions (Fig. 1B and C). Despite an initial (0–2 min) decrease in m^6^A signal at DNA lesions, treatment with a small-molecule PARG inhibitor (PARGi), which prevented PAR degradation, resulted in a slight increase of m^6^A signal 6–8 min after irradiation (Fig. 1D). We confirmed the impact of PARPi on PAR levels through immunofluorescence before and after H_2_O_2_ treatment (Fig. 1E).

To further study the role of PAR in m^6^A accumulation, we investigated m^6^A accumulation upon UV micro-irradiation in PARP1 knock-out cells (ΔPARP1). Notably, ΔPARP1 cells displayed low levels of m^6^A enrichment upon DNA damage (Fig. 1B and C). These results might be due to the involvement of other PAR polymerases, such as PARP2, or the nonspecific binding of the m^6^A antibody to nucleic acids upon induced DNA damage [60], as m^6^A signal was also detected in cells lacking the catalytic methyltransferase METTL3 upon UVC irradiation (Supplementary Fig. S1). However, the absence of m^6^A enrichment when using PARPi and the low m^6^A signal observed in ΔPARP1 cells suggest a direct correlation between PARP1 activity, PAR levels and m^6^A accumulation at DNA lesions.

### Recruitment of the METTL3/14 complex to DNA lesions depends on PAR

**Figure 2.**
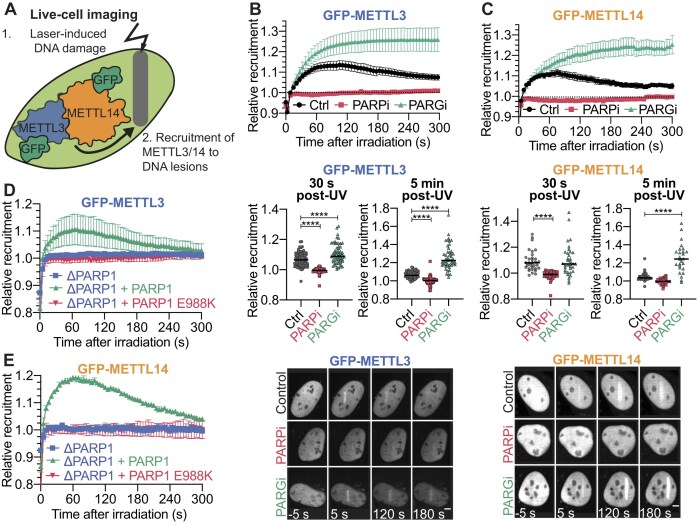
PAR dynamics drives recruitment of METTL3/14 to DNA damage sites. (**A**) Diagram of micro-irradiation experiments. Only one fluorescently tagged protein was expressed at a given time. Recruitment kinetics measured over 5 min (*left*), relative recruitment 30 s (*center*) or 5 min (*right*) after micro-irradiation of GFP-METTL3 **(B)** and GFP-METTL14 **(C)** in the presence or absence of PARPi or PARGi. More than 30 nuclei were analyzed in three independent replicates. For left panels, Data shown as mean ± SEM normalized to pre-damage GFP intensity at micro-irradiation sites. For center and right panels, each data point represents a single cell and conditions were compared with one-way ANOVA, using Dunnett’s test to compare each treatment against control **** *P*< .0001. Recruitment kinetics of GFP-METTL3 **(D)** and GFP-METTL14 **(E)** in ΔPARP1 cells, and ΔPARP1 cells transfected with either PARP1 WT or PARP1 E988K catalytic mutant measured over 5 min after micro-irradiation. More than 30 cells were analyzed per condition from three independent experiments. Size bar represents 5 μm. Data shown as mean ± SEM normalized to pre-damage GFP intensity at micro-irradiation sites.

The robust increase in m^6^A levels at DNA damage sites raises the possibility that the enzyme responsible for this RNA modification may be recruited to such sites in response to PARP1 activation. To investigate METTL3 dynamics at DNA damage sites, we generated a stable U2OS cell line expressing GFP-METTL3. Additionally, we examined METTL14 dynamics by transiently expressing GFP-METTL14 in U2OS cells. We monitored the recruitment kinetics of METTL3 and METTL14 at DNA damage sites using live-cell imaging following micro-irradiation (Fig. 2A). We observed that both GFP-METTL3 and GFP-METTL14 transiently accumulated, peaking at 60 s after irradiation (Fig. 2B and C and Supplementary Fig. S2A, *left*). This rapid accumulation resembles the dynamics of PARP1 recruitment after micro-irradiation, confirmed by co-expressing GFP-METTL3 and mCherry-PARP1 (Supplementary Fig. S2A, *right*), as well as the kinetics of the PAR-binding chromatin remodeler ALC1 [46, 48]. This contrasts with remodelers that are recruited with slower recruitment kinetics, such as CHD4 and CHD3 [61], which recruit to induced lesions following the initial wave of PAR-promoted chromatin reorganization [61].

Next, blocking the synthesis of PAR with the PARPi olaparib abolished the recruitment of METTL3 and METTL14 to DNA damage sites at both 30 s and 5 min following micro-irradiation (Fig. 2B and C). Conversely, preventing PAR degradation using PARGi, which increased nuclear PAR levels after damage, enhanced METTL3 recruitment at 30 s and 5 min after irradiation (Fig. 2B, *center*). Interestingly, initial recruitment of METTL14 was unaffected by PARGi (Fig. 2C, *center*), but increased METTL14 signal was observed 5 min after irradiation (Fig. 2C, *center*). These findings suggest a PAR-driven recruitment and retention of the m^6^A methyltransferase complex to DNA damage sites. Notably, the demethylase FTO did not visibly recruit to micro-irradiated regions (Supplementary Fig. S2B), indicating that not all components of the canonical m^6^A machinery respond to DDR-driven PARylation. Instead, the heterodimer complex rapidly recruits to DNA damage regions, likely to establish m^6^A marks at or near the induced DNA damage sites.

We validated the role of PAR in METTL3/14 recruitment by assessing their recruitment in ΔPARP1 cells. Without PARP1, neither METTL3 nor METTL14 recruited to micro-irradiation sites (Fig. 2D and E). Reintroducing wild-type PARP1 rescued the recruitment of both methyltransferases, whereas transfecting the catalytic mutant PARP1 E988K did not restore METTL3 or METTL14 recruitment (Fig. 2D and E). Additionally, the canonical substrate of METTL3/14, RNA, also influenced METTL3/14 kinetics at DNA lesions, as nascent transcription inhibition with α-amanitin (αAm) or Actinomycin D (ActD) reduced METTL3 (Supplementary Fig. S2C, *left*) and METTL14 (Supplementary Fig. S2C, *right*) recruitment. Overall, our results indicate that METTL3 and METTL14 recruitment is governed by PAR levels and represents an early event, with kinetics that mirror the dynamics of PAR synthesis and removal at damage sites.

### PAR and RNA interacts *in vitro* with recombinant METTL3/14

**Figure 3.**
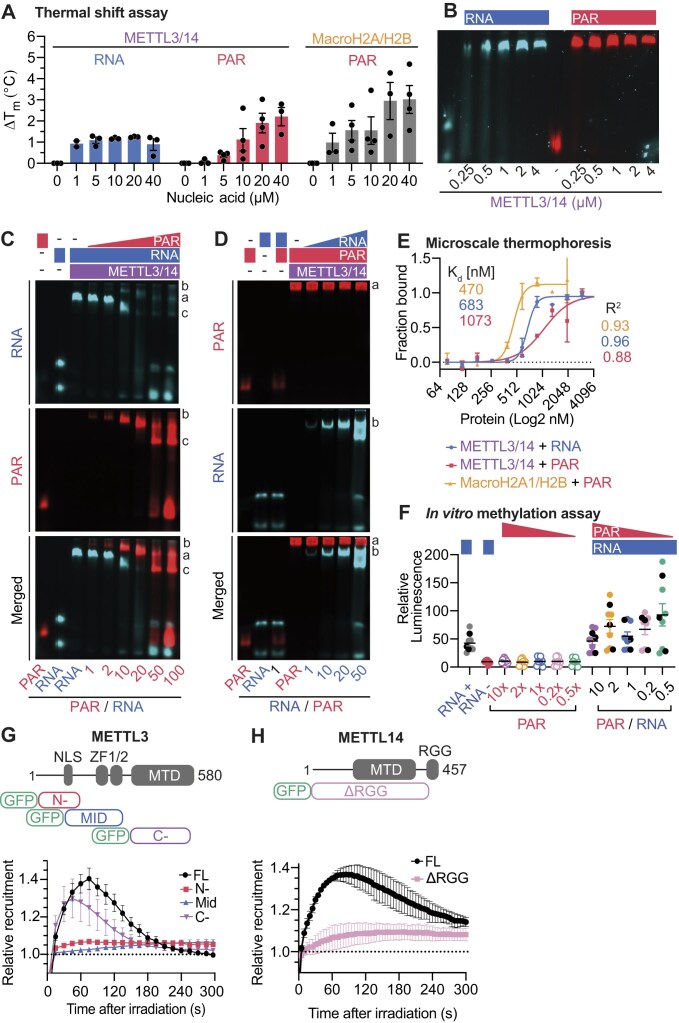
METTL3/14 can simultaneously bind PAR and RNA *in vitro*. (**A**) TSA for METTL3/14 in the presence of RNA (*left*) or RNA (*center*), and MacroH2A.1/H2B histone in the presence of PAR (*right*). Bar chart showing the changes in melting temperature (T_m_) denoting increase of thermal stability. Data shown as mean of 3–4 replicates ± SEM. (**B**) Electrophoretic mobility shift assay (EMSA) of METTL3/14 with 10 nM Cy3-RNA or AlexaFluor™ 647-PAR. (**C**, **D**) Competitive EMSA of METTL3/14 binding to RNA or PAR; METTL3/14 is incubated with RNA first and increasing concentrations of PAR are added to the reaction **(C)** or incubated with PAR first and then increasing concentrations of RNA. **(D)**. Representative images of two replicates. (**E**) Analysis of METTL3/14 mobility in the presence of PAR and RNA using microscale thermophoresis (MST). Data shown as mean of 2–4 replicates ± SEM. *K*_D_ using Hill slope model. Goodness of the fit is shown as R^2^ values. (**F**) Luminescent assay (MTAse-Glo™) to assess methylation activity of METTL3/14 in the presence of PAR, RNA or RNA + PAR at different PAR/RNA molar ratios. Data are mean of 3 experiments ± SEM. (**G**) Recruitment kinetics of METTL3 full length (FL) or truncation mutants as depicted in the diagram. (**H**) Recruitment kinetics of METTL14 FL or METTL14 ΔRGG. More than 16 cells were analyzed per condition from two independent experiments. Data shown as mean ± SD normalized to pre-damage GFP intensity at micro-irradiation sites.

Our data suggest that both PAR and RNA directly and/or indirectly contribute to recruiting METTL3/14 to DNA damage lesions. To delve deeper into the potential interaction of the nucleic acid PAR with METTL3/14 *in vitro*, we employed Nano differential scanning fluorimetry (nanoDSF). This technique enables a quantitative assessment of the thermal stability of purified METTL3/14 complex in the presence of these polymers. We utilized an RNA probe consisting of 4 tandem repeats of the “DRACH” motif, known for binding METTL3/14 [17, 62, 63]. The presence of RNA led to a slight increase of ∼1.0°C in the melting temperature (ΔT_m_) of METTL3/14 (Fig. 3A, *left*). In contrast, when METTL3/14 was incubated with PAR, a concentration-dependent increase in T_m_ of up to >2.0°C was observed (Fig. 3A, *center*), suggesting PAR-driven protein stabilization, consistent with direct binding. For comparison, we used purified MacroH2A.1.1/H2B as a control for PAR binding and observed a dose-dependent T_m_ increase of up to 3.0°C (Fig. 3A, *right*). The normalized first derivative fluorescent traces from a representative experiment are shown in Supplementary Fig. S3A (*top panel*), illustrating the T_m_ increase as a shift of the curve towards higher temperatures. A summary of T_m_ changes is provided in Supplementary Fig. S3A (*bottom panel*). METTL3/14 may thus directly bind PAR.

To gain deeper insights into how METTL3/14 interacts with RNA and PAR, we validated the *in vitro* binding of PAR to METTL3/14 using EMSA (Fig. 3B) and slot blot analysis (Supplementary Fig. S3B). Further, we performed competitive EMSAs for PAR and RNA binding to METTL3/14 by incrementally increasing the concentration of one nucleic acid to outcompete the other. When we added PAR to RNA-bound METTL3/14 complexes (marked as “a” in Fig. 3C), we noted the appearance of METTL3/14-PAR species (labeled “b” in Fig. 3C). At high concentrations of PAR, there was partial displacement of RNA, resulting in a mobility shift of the protein-nucleic acid complexes, indicated as “c” in Fig. 3C. Conversely, when RNA was used to outcompete PAR-METTL3/14 complexes (“a” in Fig. 3D), the formation of METTL3/14-RNA species was evident (“b” in Fig. 3D). However, unlike PAR, even at high concentrations, RNA was unable to displace the METTL3/14-PAR interaction, as no free PAR was detected under these conditions. Our data confirm that the METTL3/14 complex can directly recognize PAR and RNA *in vitro*. Potentially, METTL3/14 may also recognize both nucleic acids concomitantly.

To determine the affinity of the METTL3/14 complex for a canonical RNA substrate and for PAR, we employed MST and determined the binding affinities (*K*_D_) of METTL3/14 for these distinct nucleic acid probes. We obtained a *K*_D_ of 0.7 μM for the RNA target (R^2^= 0.96) and of 1.1 μM for PAR (R^2^= 0.88) (Fig. 3E). MacroH2A.1/H2B was used as a positive control for PAR binding, revealing a similar binding affinity of 0.5 μM (R^2^= 0.93). Our data indicate that the METTL3/14 complex can directly bind the related but structurally distinct nucleic acids RNA and PAR *in vitro* with similar affinity. PAR binding to METTL3/14 displays a similar affinity to that of canonical (oligo-/poly) ADP-ribose-binding domains, such as the macrodomain of the histone variant macroH2A.1.1. Our biochemical results identify the METTL3/14 complex as a PAR binder.

### Cellular interaction of METTL14 with PAR

To further explore the interaction between METTL3/14 and PAR in living cells, we employed a modified LacO-based colocalization assay. This technique involved expressing the PAR-binding macrodomain of MacroH2A.1.1 fused to LacR in U2OS 263 cells that have been engineered to contain multiple LacO repeats, allowing for the specific tethering the PAR-reader to a distinct genomic locus [64, 65]. After sensitizing our cells with BrdU and treating with PARGi, we induced high level of PARylation via laser micro-irradiation and tracked the recruitment of GFP-tagged PARP1and METTL14 to the tethered MacroH2A.1.1 bait protein. Consistent with previous reports [49], PARP1 enriched at the LacO array bound by the LacR-macrodomain. METTL14 also accumulated to the tethered PAR-reading macrodomain (Supplementary Fig. S3C), and its accumulation was reduced in the presence of PARPi (Fig. 3D, *right*). This implies that METTL14 interacts with either PAR and/or PARylated proteins within the cellular context. We further tested the interaction between PARylated PARP1 and METTL14 by tethering the methyltransferase to the LacO array, specifically by co-transfecting cells with LacR-METTL14, mCherry-METTL3, and GFP-PARP1. METTL3 readily enriched on the tethered METTL14 at the LacO array, as expected (Supplementary Fig. S3D). Upon DNA damage induction by laser-microirradiation, we observed a mild enrichment of PARylated PARP1 to the tethered METTL3/14 heterodimer in around 50% of the cells that were irradiated (Supplementary Fig. S3D and Supplementary Videos). We thus conclude that tethered METTL3/14 can recognize PARylated PARP1 in a cellular context.

To further characterize the interaction of METTL3 and METTL14 with PAR *in cellulo*, we generated constructs expressing truncated versions of the methyltransferases. We dissected METTL3 into three fragments—the N-terminal region that harbors the nuclear localization signal, the middle region which contains two zinc finger domains (ZF1/2), and the C-terminal region. All constructs were expressed in the nuclei albeit at different levels of expression. Only the C-terminal region, which contains the active methyltransferase domain of the METTL3 enzyme, recruited to micro-irradiation sites (Fig. 3G). In contrast to METTL3, METTL14 contains an RGG domain that is known to mediate RNA-binding [66, 67]. This protein domain is also known to bind to PAR [68, 69]. In the absence of the RGG domain, METTL14 did not recruit to DNA lesions (Fig. 3H). Importantly, this region is also required for methylation of RNA *in vitro*. In conclusion, the RGG domain was necessary for the recruitment of METTL14, while the MTAse domain of METTL3 was sufficient to recruit the protein to induced DNA lesions. These results indicate that the RGG domain and the MTAse domain of the METTL3/14 complex contribute to the recruitment of the complex to sites of active PAR synthesis.

### PAR does not affect the catalytic activity of METTL3/14 *in vitro*

The high structural similarities between PAR, a polymer of adenosine nucleotides, and RNA, as well as the biochemical binding of PAR by METTL3/14, led us to test whether PAR affects the catalytic activity of METTL3/14. We conducted *in vitro* methylation assays using a bioluminescence-based approach. In these assays, METTL3/14 was mixed with RNA and PAR at various RNA:PAR molar ratios, ranging from 1:0.5 to 1:10. As a negative control, we used an RNA oligo with tandem repeats of the “DRACH” motif, where adenosine was substituted with uracil (RNA-), alongside PAR at the same concentrations as in the RNA:PAR reactions. While DRACH-containing RNA was a good methyltransferase substrate for METTL3/1, PAR did not appear to be a substrate for METTL3/14 *in vitro*, nor did we observe significant changes in the luminescence signal across RNA samples containing increasing concentrations of PAR (Fig. 3F). Our results indicate that PAR did not influence METTL3/14 catalytic activity.

Collectively, we observed direct interaction between METTL3/14 and PAR. Notably, high PAR concentrations partially displaced RNA binding, but could not completely outcompete it. Conversely, RNA did not displace PAR binding, despite its higher affinity for the METTL3/14 complex. This might be due to PAR and RNA interacting with multiple domains of the METTL3/14 complex, allowing the co-existence of PAR-METTL3/14-RNA species. However, the interaction between METTL3/14 and PAR did not affect the complex’ catalytic activity, implying that PAR may rather serve as a scaffold for METTL3/14 to methylate RNA.

### METTL3 affects transcription and DNA repair

UV-induced photolesions can be repaired either via the GG-NER pathway or the TC-NER. Considering that m^6^A modification of RNA transcripts occurs co-transcriptionally [70, 71], and that METTL3 co-immunoprecipitated RBP1, the largest subunit of RNA Polymerase II (Supplementary Fig. S4A), we next aimed to explore the potential role of METTL3 in TC-NER.

**Figure 4.**
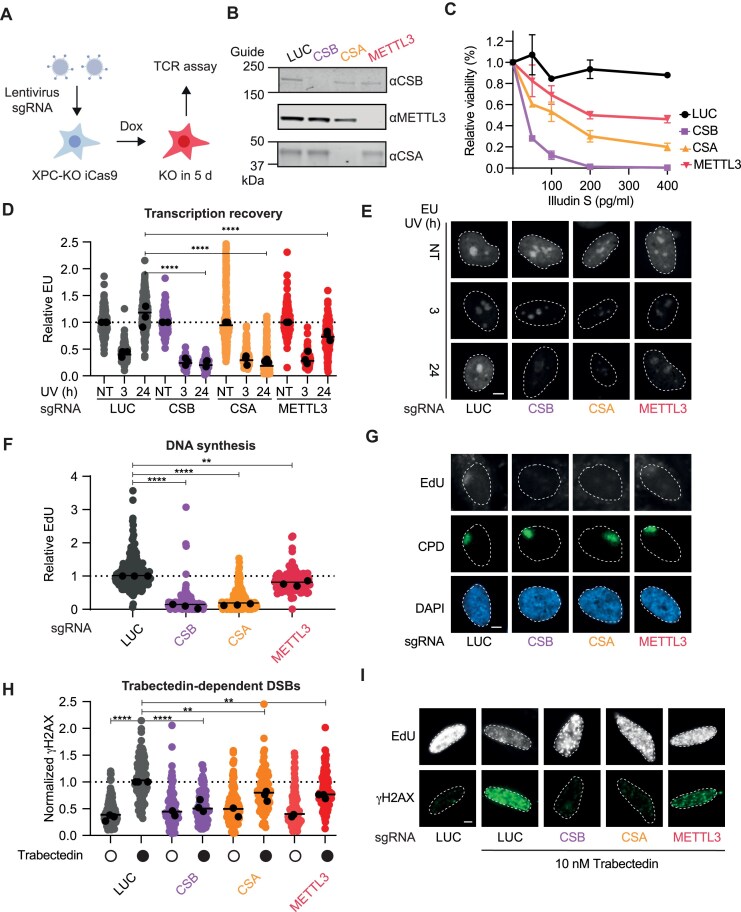
METTL3 deficiency affects transcription coupled-repair. (**A**) Lentiviral Acute KO system model in hTERT-RPE1TetOn iCas9 Puromycin/p53-dKO cells lacking XPC. (**B**) Western blot of hTERT-RPE1 cells deficient in XPC expressing guide sequences targeting CSB, CSA, and METTL3. (**C**) Cell viability assay in hTERT-RPE1 XPC-KO cells. Quantification shows the percentage of relative cell viability after 7 days of Illudin S treatment. Cells expressed guide sequences targeting luciferase (LUC), CSB, CSA, and METTL3 and relative viabilities were compared to the untreated control. Lines represent the SEM. (**D**) Quantification of RRS assay for to read-out active transcription recovery by EU-incorporation after UV irradiation in hTERT-RPE1 cells deficient in XPC. The cells expressed guide sequences targeting CSB, CSA, and METTL3. Data points from three independent experiments are shown individually, with means depicted as points near the bar representing the median of all points. Axis scale cut at 0–2.5. (**E**) Representative immunofluorescence images of the RRS assay in hTERT-RPE1 XPC-KO cells expressing guide sequences targeting LUC, CSB, CSA, and METTL3. Size bar represents 5 μm. (**F**) Quantification of DNA synthesis by the TCR-UDS assay in hTERT-RPE1 XPC-KO cells expressing guide sequences targeting LUC, CSB, CSA, and METTL3. Data points represent normalized EdU values in damaged areas marked by CPDs in the nucleus. All data points from three independent experiments are shown individually, with medians depicted as black points and the bar representing the median of all points. Axis scale cut at 0–4. (**G**) Representative immunofluorescence images of the TCR-UDS assay in hTERT-RPE1 XPC-KO cells expressing guide sequences targeting LUC, CSB, CSA, and METTL3. Size bar represents 5 μm. (**H**) Quantification of the Incision assay measuring trabectedin-dependent DSBs via _γ_H2AX nuclear intensities. Data points represent normalized γH2AX intensities to the LUC median supplemented with 10 nM trabectedin. All data points from three independent experiments are shown individually, with medians depicted as black points and the bar representing the median of all points. Axis scale cut at 0–2.5. (**I**) Representative immunofluorescence images of the Incision assay in hTERT-RPE1 XPC-KO cells expressing guide sequences targeting LUC, CSB, CSA, and METTL3.Size bar represents 5 μm.

To do this, we established a method to conditionally knockout METTL3 in RPE1 cells. We used XPC-deficient RPE1 cells, which are specifically defective in GG-NER, enabling us to study the role of METTL3 only in TC-NER. To this end, we engineered doxycycline-inducible Cas9 (iCas9) cells, which were lentivirally transduced with a nontargeting luciferase (LUC) control sgRNA, or with sgRNAs targeting either METTL3 or the TC-NER factors CSB and CSA. At 5 days after acute knockout, we induced DNA damage and monitored its repair in a manner that is strictly dependent on TC-NER [72]. A schematic representation is shown in Fig. 4A. Depletion of target proteins relative to sgLUC negative control was assessed by western blot (Fig. 4B) and sequencing (Supplementary Fig. S4B). Using this system, we assessed the sensitivity of ΔMETTL3 cells to Illudin S, a compound known to induce lesions recognized by TC-NER [50, 73]. In the absence of METTL3, cells were sensitive to Illudin S (Fig. 4C), albeit to a lesser extent compared to knockout of either CSA or CSB. Consistently, recovery of transcription after UV irradiation was reduced in METTL3-deficient cells compared to cells transduced with a nontargeting sgRNA (Fig. 4D and E). This phenotype was also observed when METTL3 was depleted by siRNA-mediated knockdown in U2OS cells (Supplementary Fig. S4C).

We further looked at the repair of DNA lesions by measuring UDS at sites of local UV damage. Using cells deficient in GG-NER combined with an optimized method, enables us to specifically capture UDS during TC-NER [56]. Consistent with their known role in TC-NER, knockout of either CSA or CSB caused a strong defect in the repair of transcription-blocking DNA lesions, evident by strongly impaired EdU incorporation at damage sites identified by immunostaining for CPD. Cells transduced with sgRNAs targeting METTL3 showed a mild decrease in EdU incorporation compared to Luc control cells (Fig. 4F and G), suggesting that METTL3 contributes to the efficient repair of DNA lesions encountered during transcription. The CPD levels of the different sgRNA-transduced cells are shown in Supplementary Fig. S4D.

To extend these findings, we treated cells with trabectedin, a chemotherapeutic that generates transcription-blocking lesions that are processed by TC-NER into DSBs [72]. When cells transduced with a nontargeting sgRNA were treated with trabectedin, we detected a marked increase in γH2AX signal specifically in replicating cells marked by EdU incorporation (Fig. 4H and I). This signal was strongly suppressed to near background levels in the CSB knockout pool, confirming this read-out is specific for TC-NER. Cells transduced with sgRNAs against METTL3 showed strongly reduced γH2AX signal upon trabectedin treatment (Fig. 4H and I), consistent with a defect in TC-NER. To better connect the METTL3 recruitment data to the functional data shown here, we conducted similar experiments using olaparib to test whether PARP1 inhibition also affects TC-NER, as observed with METTL3 loss. Inhibition of PARP1 alone reduced transcription recovery to a level comparable to METTL3 depletion in XPC-deficient cells (Supplementary Fig. S4E and F). Furthermore, UDS and γH2AX signals were decreased following PARPi treatment (Supplementary Fig. S4G and I). These findings suggest that the absence of PARP1 impairs TC-NER to a similar extent as METTL3 loss in XPC-deficient RPE1 cells. Collectively, these findings underscore METTL3’s role in the DDR, highlighting its impact transcription-coupled DNA repair and transcription recovery following DNA damage.

### PARP and METTL3 inhibitors together decrease cell proliferation

In the clinic, PARPi are approved for treating several solid tumors, including breast, prostate, and ovarian cancer, but are also being investigated preclinically in hematological malignancies (reviewed in [74]). Their application in the context of deficiencies in homologous recombination-mediate DNA repair represents the first synthetic lethal therapy, as cells harboring BRCA1 or BRCA2 mutations, for example, are particularly sensitive to PARPi.

METTL3 on the other hand is a critical player acute myeloid leukemia (AML) [75], which has led to the development of METTL3 inhibitors, some of which are undergoing Phase 1 clinical trials (Identifier: NCT05584111). Beyond the interaction between METTL3 and PARP1 at DNA lesions, there is growing evidence suggesting a role for METTL3 in DNA repair through homologous recombination [42, 76], and regulation of DDR transcripts following DNA damage [40, 77, 78]. Therefore, we hypothesized that combining METTL3 and PARP inhibitors could synergize in treating cancer cells.

**Figure 5.**
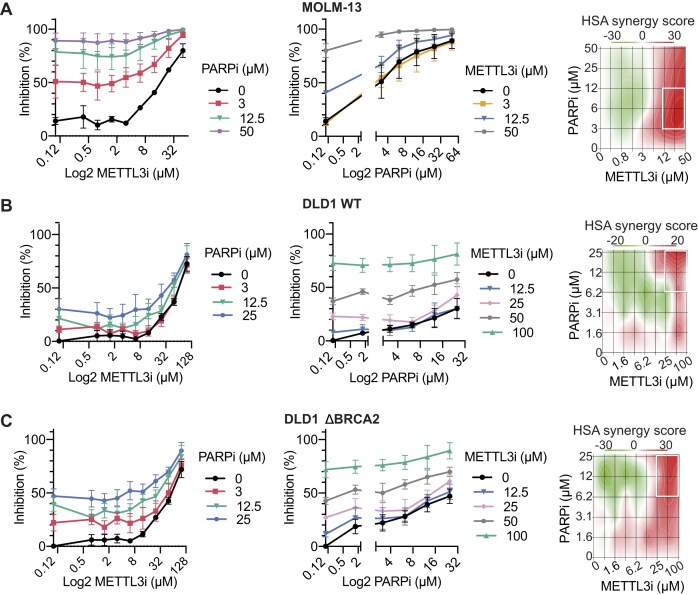
Combination of METTL3 and PARP inhibitors hinders the proliferation of cancer cells. (*Left* and *center*) Proliferation inhibition of MOLM-13 AML cells **(A)**, colorectal adenocarcinoma BRCA-proficient DLD1 WT **(B)** BRCA2 deficient **(C)** cells after treatment with the PARPi olaparib and/or METTL3i STM2457 at different concentrations. Data are depicted as the mean of three biological replicates, each containing two technical replicates ± SEM. Viability was measured using CellTiter-Glo^®^ and percentage inhibition as the inverse ratio of the luminescence of the sample and a live control, corrected for background signal. (*Right*) Synergy score calculated using based on the HSA reference model using SynergyFinder 2.0. Deviations between observed and expected responses with positive and negative values denote synergy and antagonism respectively. With rectangle denotes the area with higher synergy.

We first explored this hypothesis in MOLM-13 cells, an AML model. We examined the growth inhibition of MOLM-13 cells treated with the METTL3i STM2457, the PARPi olaparib, or a combination of both. After 72 h of continuous treatment, cell viability was measured using a luminescent-ATP-based assay. Consistent with previous findings [21], MOLM-13 cells were sensitive to METTL3 inhibition (Fig. 5A, *left*), compared to unrelated cell types (DLD1, Fig. 5B and C, *left* and MDA-MB-231 and SUM149PT, Supplementary Fig. S5A and B, *left*). Notably, combining METTL3i with PARPi in MOLM-13 cells resulted in a robust increase in inhibitory potency, as evidenced by the shifts in the dose-response curves (Fig. 5, *center*). The expected drug combination responses were calculated based on the HSA model using SynergyFinder [58]. This model assumes that the expected combination effect is the maximum of the single drug responses at the corresponding concentrations [79]. Deviations between expected and observed responses denote synergy for scores above 10, and additive interaction for scores between 0 and 10. Based on this model, the combination of PARPi and METTL3i exhibited synergistic interaction at the areas of highest synergy score (Fig. 5A and Supplementary Fig. S5A–C), albeit at high concentrations of the individual drugs, where the off-target effects cannot be ruled out.

To assess whether the METTL3i-PARPi interaction depends on the BRCA status of the cells, we tested this combination approach in DLD1 WT and isogenic DLD1 BRCA2 knockout cells. While additive interaction was observed at the highest interaction area on DLD1 WT cells, cells deficient in BRCA2 exhibited synergistic interaction at higher concentrations (Fig. 5B and C, *right*, and Supplementary Fig. S5C).

Additionally, we evaluated the effect of this inhibitor combination on the BRCA1/2 proficient triple negative MDA-MB-231 and the BRCA1-mutant triple negative SUM149PT cell lines. In these cell lines, similar to MOLM-13, the combination of PARPi with METTL3i enhanced the proliferation inhibition to levels where complete inhibition of cellular growth was observed (Supplementary Fig. S5A and B). Synergistic drug interaction was observed for both cell lines, independent of BRCA1 status (Supplementary Fig. S5A–C). These data indicate that METTL3i and PARPi combination enhances cell growth inhibition.

## Discussion

One of the initial steps in the DDR is the recognition of DNA lesions by PARP1, which triggers a rapid, transient burst of PARylation. PAR dynamics are crucial in the DDR, aiding chromatin relaxation, activating downstream factors via post-translational modification, and serving as a scaffold for DDR factors. Previous research indicated m^6^A accumulation at micro-irradiation sites and its reduction with PARPi [40]. However, the direct link between PAR-driven METTL3/14 accumulation and whether METTL3/14 recognizes PAR was not clear. In our study, we explored the interactions between PAR and m^6^A by providing kinetic detail on how PAR prompts METTL3/14 complex recruitment to DNA lesions.

Through micro-irradiation and live-cell imaging, we noted rapid, transient accumulation of METTL3 and METTL14 at DNA lesions. This recruitment depended on PAR dynamics, as modifying PAR levels with PARPi and PARGi either inhibited or sustained METTL3/14 at the damage sites, respectively. PARP1-deficient cells further affirmed that METTL3/14 recruitment relies on PAR production at DNA lesions. While laser micro-irradiation can cause DNA breaks and METTL3/14 can methylate DNA *in vitro* [43], our data suggest that DNA breaks alone are insufficient for METTL3/14 recruitment and that METTL3/14 recruitment to micro-irradiation sites is stimulated by PAR synthesis. Additionally, inhibiting RNA synthesis impacted the recruitment of the methyltransferase dimer. Future studies are essential to further unravel the complex interaction between PAR and RNA at DNA damage sites and its occurrence in homeostatic contexts, such as at active gene transcription sites.

Building on previous observations [40], we confirmed a role of METTL3 in DNA repair, particularly in TC-NER. Using a RPE1 cells deficient in GG-NER in combination with inducible acute depletion of METTL3, we confirmed that the absence of METTL3 delays transcriptional restart after UV damage. Moreover, METTL3 depletion resulted in a mild sensitivity against Illudin S. The reduced UDS at DNA photolesions together with the decreased γH2AX signal after trabectedin treatment suggest a role of METTL3 in TC-NER. Our observations further validate previous genome-wide screenings mapping genetic susceptibilities against DNA damage agents, where METTL3 depletion resulted in UV sensitivity while conferring resistance against trabectedin [80]. Similar sensitivities were reported for TC-NER factors, such as ELOF1 [50, 80].

Since identifying METTL3 as a key oncogenic factor in AML [75], there has been significant progress in developing METTL3 inhibitors [21], with the first clinical trial currently underway. Given the clinical use of PARPi, we explored the potential of combining METTL3i with PARPi in cultured cancer cells. Our experiments showed a synergistic anti-proliferative effect in cells derived from AML (MOLM-13) and triple-negative breast cancer BRCA-proficient (MDA-MB-231) and BRCA-deficient (SUM194PT), and additive effect in colorectal adenocarcinoma (DLD1) cells, when these drugs were combined. Similar observations have been recently reported [78, 81]. These data support the further analysis of efficacy in different cancer models and suggests an additive, and potential synergistic drug effect, independent of BRCA-proficiency status.

Proteomic analysis reveals significant enrichment of RBPs among PAR binders [82]. RBPs like FUS, EWS and DHX9 [38, 83] are recruited to micro-irradiation sites in a PAR-dependent manner. However, the biochemical resemblance between PAR and RNA raises questions about their interaction specificity and the mechanisms of selective interaction between PAR and RNA for RBPs. Our *in vitro* data are consistent with direct binding of METTL3/14 to PAR polymers, with high PAR concentrations partially displacing RNA binding, but not outcompeting it. Conversely, RNA could not displace PAR in our hands. Moreover, deletion of the RGG domain of METTL14 prevented the recruitment of the methyltransferase to DNA damage sites, while the MTAse domain of METTL3, but not of METTL14, was sufficient to recruit to DNA lesions, possibly through PAR interaction. Nonetheless, previous structural studies highlight the cooperative contribution of the structural domains of the two subunits towards the catalytic activity of the complex. For instance, while in our hands the removal of the zinc-binding motifs did not affect the recruitment of METTL3 to DNA lesions, point mutations in this region disrupt the catalytic activity of the complex *in vitro* [14]. This highlights the need to study the contribution of each domain in towards RNA and PAR binding in the context of the full-length complex.

We observed no difference in METTL3/14’s catalytic activity in the presence of PAR, yet a tethered PAR-binding macrodomain enriched METTL3/14, leading us to propose that PAR and RNA may not compete for METTL3/14 binding. Instead, PAR likely serves as a scaffold, guiding METTL3/14 to specific nuclear locations and enabling its targeted enzymatic activity. Conversely, we also observed that tethered METTL3/14 can recruit PARylated PARP1 in the cellular context. Our findings thus raise the possibility that the interaction between PAR, RNA and METTL3/14 may also take place in other biological contexts, for example during transcription or in the presence of DNA:RNA species. Moreover, understanding its biological implications can help dissect the overlapping RBP landscape from the PARylome, opening potential therapeutic venues.

In summary, our work provides insights into PAR interacting with METTL3/14, driving the recruitment of METTL3/14 to DNA lesion sites and PAR serving as a scaffold without affecting the activity of the methyltransferase complex. Our data identify a genetically distinct role of METTL3 in TC-NER, locating METTL3 at the intersection between PAR, RNA, and DNA repair. Furthermore, the combination of PARPi and METTL3i effectively inhibits the proliferation of cultured cancer cells, hinting at the potential that such as drug combination may play in suppressing tumor cell growth. Assuming that combining these two therapeutic agents does not exacerbate the established toxicity profiles for the individual monotherapies, our results could pave the way for innovative combination treatments, potentially enabling the targeting of new cancer indications.

## Supplementary Material

gkaf244_Supplemental_Files

## Data Availability

The data underlying this article are available in the article and in its online supplementary material.
