# Supplementary material for: Poly-(ADP-ribose) serves as a scaffold for the methyltransferase METTL3/14 complex in the DNA damage response
Source: Nucleic Acids Res. 2025 Apr 12;53(7):gkaf244. doi: 10.1093/nar/gkaf244 (PMC11992677; doi:10.1093/nar/gkaf244)
Supplement: gkaf244_Supplemental_Files [file gkaf244_supplemental_files.zip › 20250308 Manuscript - Supplementary Data.pdf]

Poly-(ADP-ribose) serves as a scaffold for the methyltransferase METTL3/14 complex in the DNA damage response

Claudia Gonzalez-Leal[1,2], Jin Cai[1,2,†], Bram A.F.J. de Groot[3,†], Andreas Wegerer[1], Julia Preisser[1], Martijn S. Luijsterburg[3] and Andreas G. Ladurner[1,2,4,*]

[1] Department of Physiological Chemistry, Biomedical Center (BMC), Faculty of Medicine, LMU Munich, 82152 Planegg – Martinsried, Germany.

[2] International Max Planck Research School (IMPRS) for Molecules of Life, Planegg-Martinsried, Germany.

[3] Department of Human Genetics, Leiden University Medical Center (LUMC), Leiden, the Netherlands.

[4] Eisbach Bio GmbH, Am Klopferspitz 19, 82152 Planegg-Martinsried, Germany.

* To whom correspondence should be addressed. Tel: +49 (89) 2180 77095; Fax: +49 (89) 2180 77093; Email: andreas.ladurner@bmc.med.lmu.de

[†] Joint Authors.

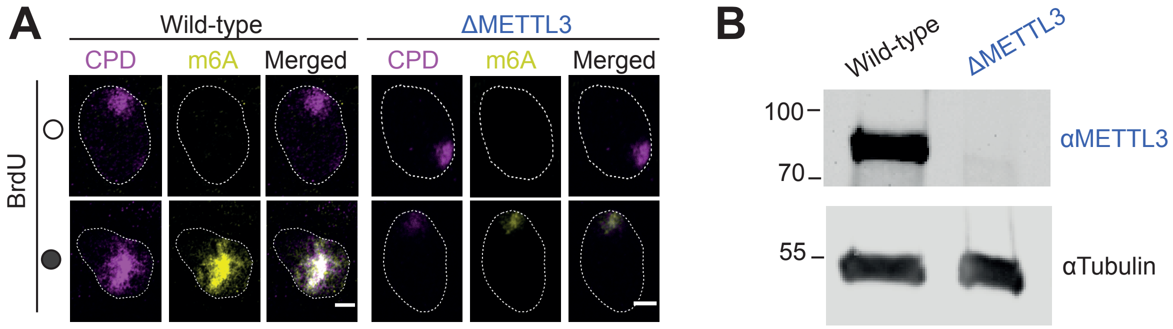

Figure S1. PAR drives the accumulation of m6A at micro-irradiation sites.
(**A**) Immunofluorescence after UVC irradiation through a micropore membrane. m6A signal is observed in BrdU-sensitized cells and it co-localizes with CPD lesions. Residual m6A signal is observed in METTL3-deficient cells. Representative images of two independent experiments with over 100 cells per condition. Size bar represents 5 µm. (**B**) METTL3 depletion in knock-out cells was confirmed by western blot.

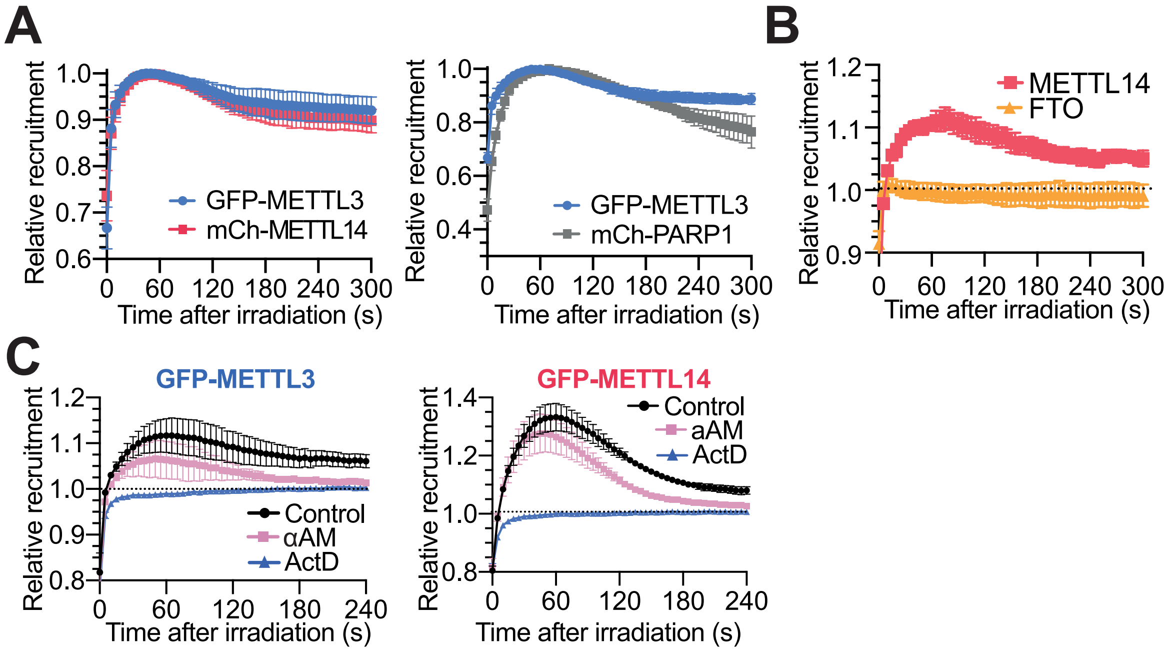

Figure S2. PAR dynamics drives the recruitment of METTL3/14 to DNA damage sites.
(**A**) Recruitment kinetics of GFP-METTL3 in cells co-expressing mCh-METTL14 (*left*) or mCh-PARP1 (*right*). Data for >16 nuclei from two independent experiments shown as mean ± SD normalized to pre-damage signal intensity at micro-irradiation sites and to peak signal intensity. (**B**) Recruitment kinetics of GFP-FTO to micro-irradiation sites. FTO does not recruit to micro-irradiation sites. GFP-METTL14 was used as a positive control. (**C**) Recruitment kinetics of GFP-METTL3 (*left*) and GFP-METTL14 (*right*) after treatment with RNA Polymerase inhibitors, α-Amanitin (αAM) and Actinomycin D (ActD). (**B-C**) Data for >30 nuclei from three independent experiments shown as mean ± SEM normalized to pre-damage GFP intensity at micro-irradiation sites.

A Thermal shift assay

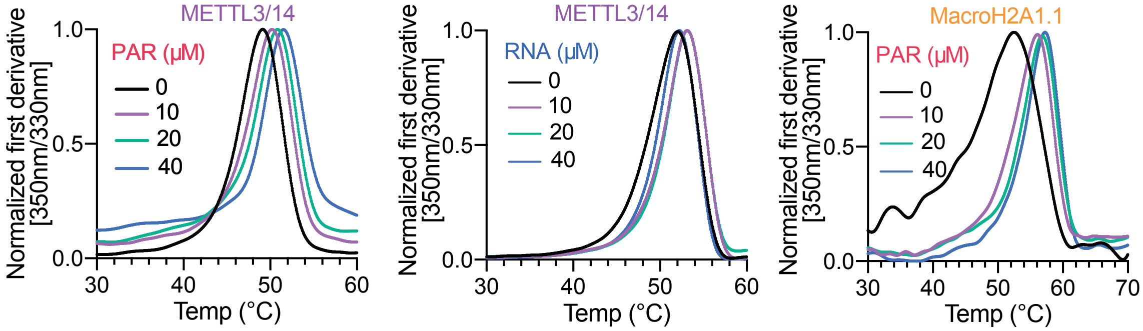

METTL3/14				MacroH2A1.1	
RNA (μM)	Mean T_m (°C) ±SEM	PAR (μM)	Mean T_m (°C) ±SEM	PAR (μM)	Mean T_m (°C) ±SEM
Apo	**49.6** ± 0.7	Apo	**49.6** ± 0.7	Apo	**49.6** ± 0.7
1	**51.2** ± 1.7	1	**49.8** ± 0.8	1	**49.8** ± 0.8
5	**50.9** ± 1.1	5	**50.0** ± 0.6	5	**50.0** ± 0.6
10	**51.0** ± 1.0	10	**50.7** ± 0.6	10	**50.7** ± 0.6
20	**51.1** ± 1.0	20	**51.5** ± 0.4	20	**51.5** ± 0.4
40	**50.7** ± 0.7	40	**52.0** ± 1.3	40	**52.0** ± 1.3

B Slot blot

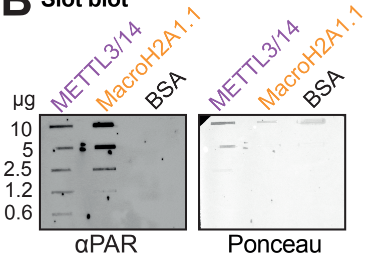

C Fluorescence-two hybrid - tethered macroH2A1.1

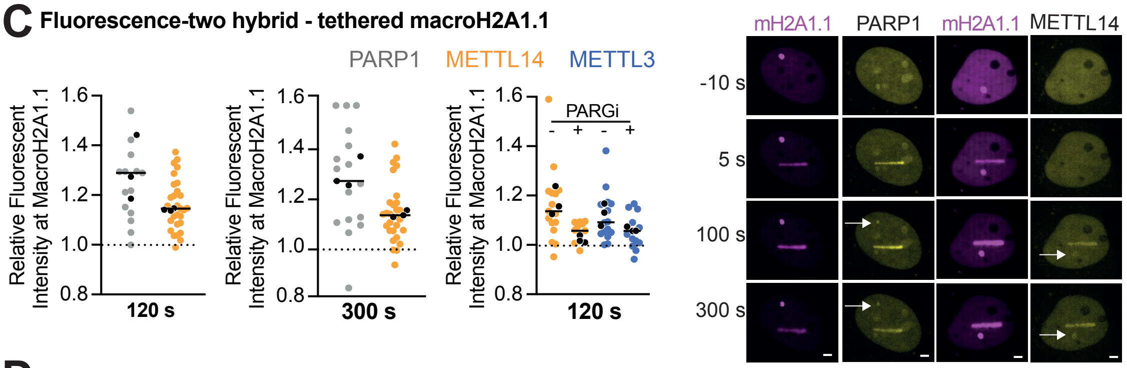

D Fluorescence-two hybrid - tethered METTL14

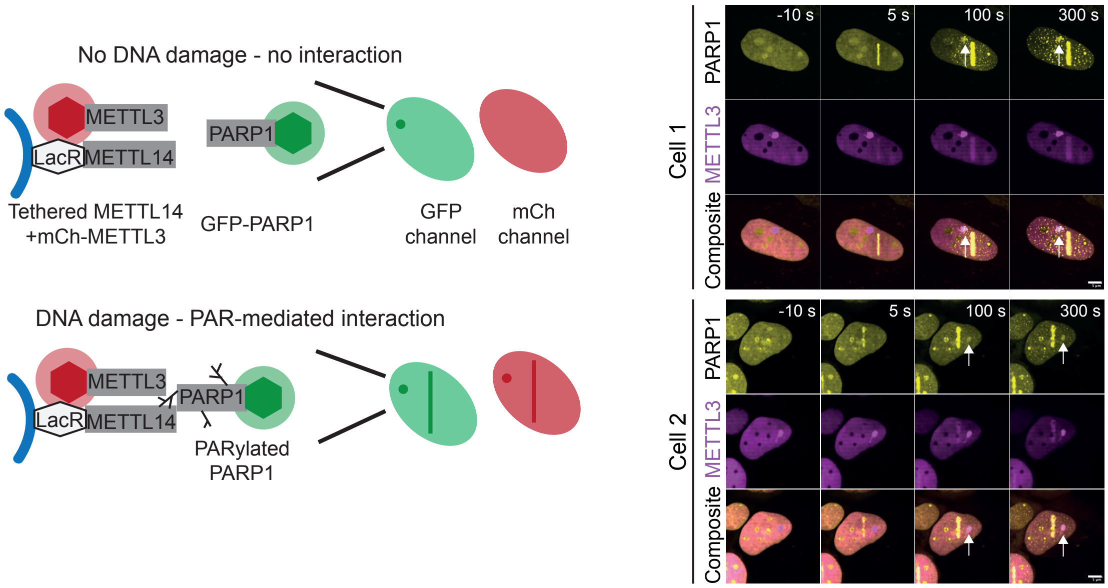

Figure S3. METTL3/14 interacts with PAR and RNA *in vitro*

(**A**) Representative traces and differences in melting temperature (bottom) of thermal shift assay measured with nanoDSF of recombinant METTL3/14 in the presence of PAR or RNA. MacroH2A.1.1/H2B was used as positive control for PAR interaction. (**B**) Slot blot of immobilized recombinant protein incubated with 1 µM PAR and developed against PAR. BSA was used as negative control and Ponceau as loading control. (**C**) Representative images and quantification of GFP-tagged PARP1 and METTL14 recruitment to the LacO array upon tethering to mChery-LacR-MacroH2A.1.1 on cells treated with PARGi (*left* and *center*) or PARGi in the presence and absence of PARPi (*right*). Treatment with PARPi reduced the accumulation of METTL14 to the tethered MacroH2A.1.1. Fluorescent intensity at the LacR was normalized against nuclear signal. The means of 3-4 biological replicates are depicted as black points, while the bar represents the median of all nuclei (>30) analyzed. Size bar represents 5 µm. (**D**) Diagram and representative images of GFP-PARP1 recruitment to the LacO array upon tethering of LacI-METTL14 and expressing mCherry-METTL3 in cells treated with PARGi. Tethered METTL14 is visualized through the formation of a LacI–METTL14/mCherry–METTL3 heterodimer. The accumulation of PARP1 upon DNA damage induction with the microirradaition laser is marked with arrows. >120 cells were analyzed. We observed PARP1 accumulation in about 51% of irradiated cells. Additional videos are provided as Supplementary Material. Size bar represents 5 µm.

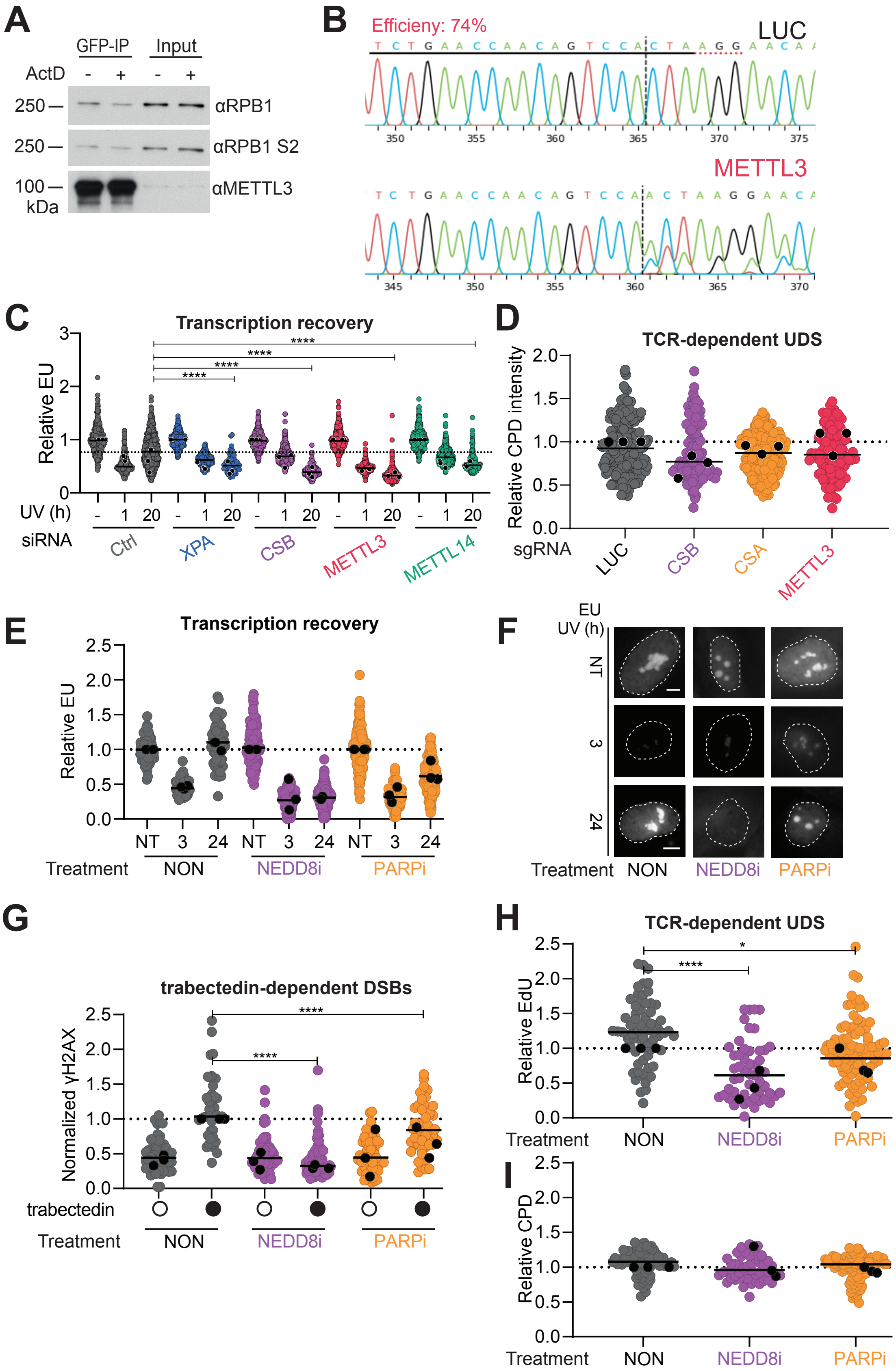
A
GFP-IP
Input
ActD
250
αRPB1
250
αRPB1 S2
100
kDa
αMETTL3
B
Efficieny: 74%
LUC
METTL3
C
Transcription recovery
Relative EU
UV (h)
siRNA
Ctrl
XPA
CSB
METTL3
METTL14
D
TCR-dependent UDS
Relative CPD intensity
sgRNA
LUC
CSB
CSA
METTL3
E
Transcription recovery
Relative EU
Treatment
NON
NEDD8i
PARPi
F
EU
UV (h)
NT
3
24
Treatment
NON
NEDD8i
PARPi
G
trabectedin-dependent DSBs
Normalized γH2AX
trabectedin
Treatment
NON
NEDD8i
PARPi
H
TCR-dependent UDS
Relative EdU
Treatment
NON
NEDD8i
PARPi
I
Relative CPD
Treatment
NON
NEDD8i
PARPi

Figure S4. METTL3 and PARP deficiency delays transcription and DNA repair.
(**A**) IP of GFP-METTL3 shows interaction with RBP1, a subunit of RNA Polymerase II. (**B**) Editing efficiency of hTERT-RPE1 cells deficient in XPC-expressing guide sequences targeting Luciferase (LUC) and METTL3. (**C**) EU-incorporation assay to assess active transcription after UV irradiation in cells depleted of the TC-NER factors XPA and CSB, as well as the methyltransferases METTL3 and METTL14. The means of three biological replicates are depicted as black points, while the bar represents the median of all points. Conditions were compared using one-way ANOVA using Tukey's test to compare each treatment against wild-type 16h, **** $p<0.0001$. Axis scale cut at 0 to 3. (**D**) Quantification of the immunofluorescence assay which shows the relative CPD intensity of hTERT-RPE1 cells deficient in XPC-expressing guide sequences targeting Luciferase (LUC), CSB, CSA and METTL3. All datapoints from three independent experiments are depicted as individual points. The medians of three biological replicates are depicted as black points, the bar represents the median of all points. Axis scale cut at 0 to 2. (**E**) Quantification of the RNA Recovery Synthesis (RRS) assay to read-out active transcription recovery by EU-incorporation after UV irradiation in hTERT-RPE1 cells deficient for XPC. Cells were treated with or without nedylation inhibitor (NEDD8i) and PARP inhibitor (PARPi). Data points from three independent experiments are shown individually, with means depicted as black points and the bar representing the median of all points. Axis scale cut at 0 to 2.5. (**F**) Representative immunofluorescence images of the RRS assay in hTERT-RPE1 XPC-KO cells treated with or without nedylation inhibitor (NEDD8I) and PARP inhibitor (PARPI). Size bar represents 5 µm. (**G**) Quantification of the Incision assay measuring trabectedin-dependent DSBs via γH2AX nuclear intensities. Data points represent normalized γH2AX intensities to the non-treated control supplemented with 10 nM trabectedin. All data points from three independent experiments are shown individually, with medians depicted as black points and the bar representing the median of all points. Conditions were compared using one-way ANOVA to compare each treatment against the non-treated, **** $p<0.0001$, **** $p<0.0001$. Axis scale cut at 0 to 2.5. (**H**) Quantification of DNA synthesis by the TCR-UDS assay in hTERT-RPE1 XPC-KO cells. Data points represent normalized EdU values in damaged areas marked by CPDs in the nucleus. Cells were treated with or without nedylation inhibitor (NEDD8i) and PARP inhibitor (PARPi). All data points from three independent experiments are shown individually, with medians depicted as black points and the bar representing the median of all points. Conditions were compared using one-way ANOVA to compare each treatment against the non-treated, **** $p<0.0001$, * $p<0.05$. Axis scale cut at 0 to 2.5. (**I**). Quantification of the immunofluorescence assay which shows the relative CPD intensity of hTERT-RPE1 cells deficient for XPC. Cells were treated with or without nedylation inhibitor (NEDD8i) and PARP inhibitor (PARPi). All datapoints from three independent experiments are depicted as individual points. The medians of three biological replicates are depicted as black points, the bar represents the median of all points. Axis scale cut at 0 to 2.5.

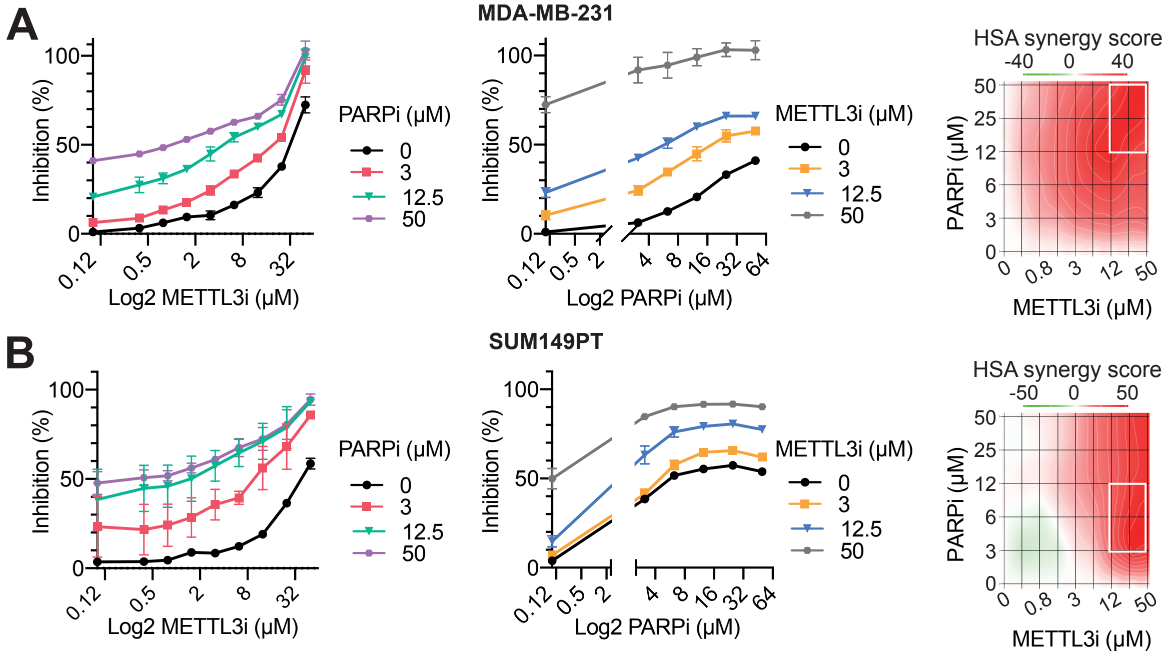

C

HSA Synergy Score

Cell line	BRCA Status	Mean ±SEM	Highest Area
**MOLM13**	**WT**	**5.6** ± 4.1	**21.4**
**MDA-MB-231**	**WT**	**19.9** ± 1.2	**34.0**
**SUM149PT**	**BRCA1 loss**	**16.8** ± 0.9	**39.0**
**DLD1 WT**	**WT**	**0.02** ± 4.1	**6.5**
**DLD1 ΔBRCA2**	**ΔBRCA2**	**3.2** ± 4.3	**14.8**

Figure S5. Combination of METTL3 and PARP inhibitors hinders the proliferation of cancer cells.

(*Left* and *center*) Proliferation inhibition of (**A**) MDA-MB-231 BRCA-proficient triple negative breast cancer cells and (**B**) SUM149PT BRCA1-mutant triple negative breast cancer after treatment with the PARPi olaparib and/or METTL3i STM2457 at different concentrations. Data is depicted as the mean of three biological replicates, each containing two technical replicates ± SEM. Viability was measured using CellTiter-Glo® and percentage inhibition as the inverse ratio of the luminescence of the sample and a live control, corrected for background signal. (*Right*) Synergy score calculated using based on HAS reference model using SynergyFinder 2.0. Deviations between observed and expected responses with positive and negative values denote synergy and antagonism respectively. With rectangle denotes the area with higher synergy. (**C**) Summary of synergy scores of the cell lines analyzed. Scores between -10 and 10 represent potential additive drug interactions, while values >10 are likely synergistic drug interactions.
